# A multi‐omics study delineates new molecular features and therapeutic targets for esophageal squamous cell carcinoma

**DOI:** 10.1002/ctm2.538

**Published:** 2021-09-26

**Authors:** Xing Jin, Lei Liu, Jia Wu, Xiaoxia Jin, Guanzhen Yu, Lijun Jia, Fengying Wang, Minxin Shi, Haimin Lu, Jibin Liu, Dan Liu, Jing Yang, Hua Li, Yan Ni, Qin Luo, Wei Jia, Wei Wang, Wen‐Lian Chen

**Affiliations:** ^1^ Cancer Institute Longhua Hospital Shanghai University of Traditional Chinese Medicine Shanghai China; ^2^ Department of Thoracic Surgery The Affiliated Tumor Hospital of Nantong University Nantong China; ^3^ Department of Pathology The Affiliated Tumor Hospital of Nantong University Nantong China; ^4^ Bio‐ID Center School of Biomedical Engineering Shanghai Jiao Tong University Shanghai China; ^5^ The Children's Hospital National Clinical Research Center for Child Health Zhejiang University School of Medicine Hangzhou China; ^6^ Hong Kong Traditional Chinese Medicine Phenome Research Center School of Chinese Medicine Hong Kong Baptist University Kowloon Tong Hong Kong China

**Keywords:** esophageal squamous cell carcinoma, fibrillarin, molecular feature, multi‐omics

## Abstract

Esophageal squamous cell carcinoma (ESCC) is a major histological subtype of esophageal cancer with inferior prognosis. Here, we conducted comprehensive transcriptomic, proteomic, phosphoproteomic, and metabolomic characterization of human, treatment‐naive ESCC and paired normal adjacent tissues (cohort 1, *n* = 24) in an effort to identify new molecular vulnerabilities for ESCC and potential therapeutic targets. Integrative analysis revealed a small group of genes that were related to the active posttranscriptional and posttranslational regulation of ESCC. By using proteomic, phosphoproteomic, and metabolomic data, networks of ESCC‐related signaling and metabolic pathways that were closely linked to cancer etiology were unraveled. Notably, integrative analysis of proteomic and phosphoproteomic data pinpointed that certain pathways involved in RNA transcription, processing, and metabolism were stimulated in ESCC. Importantly, proteins with close linkage to ESCC prognosis were identified. By enrolling an ESCC patient cohort 2 (*n* = 41), three top‐ranked prognostic proteins X‐prolyl aminopeptidase 3 (XPNPEP3), bromodomain PHD finger transcription factor (BPTF), and fibrillarin (FBL) were verified to have increased expression in ESCC. Among these prognostic proteins, only FBL, a well‐known nucleolar methyltransferase, was essential for ESCC cell growth in vitro and in vivo. Furthermore, a validation study using an ESCC patient cohort 3 (*n* = 100) demonstrated that high FBL expression predicted unfavorable patient survival. Finally, common cancer/testis antigens and established cancer drivers and kinases, all of which could direct therapeutic decisions, were characterized. Collectively, our multi‐omics analyses delineated new molecular features associated with ESCC pathobiology involving epigenetic, posttranscriptional, posttranslational, and metabolic characteristics, and unveiled new molecular vulnerabilities with therapeutic potential for ESCC.

## Graphical abstract

1. A new molecular feature of ESCC that involves both active posttranscriptional and posttranslational regulation was unveiled.

2. ESCC‐related signaling and metabolic pathways, networks among omics data, and common cancer/testis antigens along with established cancer drivers and kinases were delineated.

3. Proteins with close linkage to ESCC prognosis were discovered, and a new prognostic protein, fibrillarin (FBL), was further validated, functionally studied, and found to correlate negatively with patient outcomes.

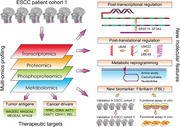

## 1 INTRODUCTION

Esophageal carcinoma (EC) is the ninth most common cancer and ranks sixth with respect to lethality in the world.^1^ In China, the morbidity and mortality of EC are ranked fifth and fourth, respectively, across all cancers.^2^ The major histological type of EC in China is esophageal squamous cell carcinoma (ESCC), accounting for approximately 90% of all EC cases.^1^ To decipher the molecular aberrations that drive ESCC tumorigenesis and progression, The Cancer Genome Atlas (TCGA) and other research teams have conducted extensive genomic, epigenomic, and transcriptomic profiling, discovering unique signatures of ESCC that contain frequent genomic amplifications of tumor‐promoting genes and those modulating cell cycle and apoptosis genes that have high mutational frequency.^3^, ^4^, ^5^, ^6^, ^7^, ^8^, ^9^ Notably, a predominant event in ESCC development is mutation and inactivation of a well‐known tumor suppressor, TP53.^4^, ^9^ Another typical hallmark of ESCC is mutation and/or genomic amplification of cell cycle kinases including cyclin D1 (CCND1) and cell division protein kinase 6 (CDK6).^3^, ^4^, ^6^ Additionally, it is common to observe mutations of genes involved in epigenetic processes and Notch/PI3K/EGFR/Hippo pathways in ESCC.^3^, ^4^, ^6^

Due to the advances in genomic study, more targeted therapies are being designed for ESCC treatment. Unfortunately, except from HER2‐positive ESCC tumors, randomized controlled trials of targeted therapies for other targets, such as EGFR and mesenchymal–epithelial transition pathways, have failed.^1^ The results of these trials demonstrate the complexity of ESCC oncogenesis and progression and demonstrate the limitation of genomic profiling alone for identifying effective curative treatments. Hence, a multi‐omics approach may provide the necessary information required to unveil more effective molecular targets for ESCC treatment.

In this study, we performed a multi‐layer omics study to characterize human, treatment‐naive ESCC tumors and paired normal adjacent tissues (NATs), aiming to delineate the mechanisms of ESCC pathobiology and unveil new therapeutic targets for precise and personalized clinical intervention.

## 2 MATERIALS AND METHODS

### 2.1 Nano‐liquid chromatography‐tandem mass spectrometry analysis

#### 2.1.1 Proteomic analysis using a data‐independent approach

Data‐dependent analysis (DDA) was performed first to generate a DDA spectral library. Fractionated and reconstituted peptides (∼1 μg each fraction) of each pooled sample were resolved using a micro‐tip C18 column (75 μm × 25 cm) packed with ReproSil‐Pur C18‐AQ, 5 μm resin (Dr. Maisch GmbH, Germany) coupled to a nanoflow HPLC Easy‐nLC 1200 system (Thermo Fisher Scientific, cat#LC140) with LC gradient rate at 250 nl/min. Two buffer solutions were used, including buffer A (a mixture of formic acid:H_2_O = 1:1000 [vol/vol]) and buffer B (a mixture of formic acid:acetonitrile = 1:1000 [vol/vol]). Pooled sample resolving was achieved with the following separation gradient: 8%–30% buffer B from 0 to 97 min; 30%–100% buffer B from 97 to 100 min; 100% buffer B from 110 to 120 min. Subsequent assays were conducted on a Q‐Exactive HF mass spectrometer (Thermo Fisher Scientific, cat#IQLAAEGAAPFALGMBFZ). Positive ion mode was used for detection. The MS1 full scan was set with a range of 300–1800 m/z, and with resolution of 60,000 at m/z 200, AGC target 3e6, and maximum IT 50 ms. A total of 20 MS2 scans were collected after the MS1 scan based on the inclusion list. The MS2 scans were acquired at resolution of 30,000 at m/z 200, AGC target 3e6, maximum IT 120 ms, activation type as HCD, and normalized collision energy at 27 eV.

Subsequently, for data‐independent analysis (DIA), 2 μg digested peptides of each case mixed with iRT standard peptides were resolved with the same instrument and buffer solutions used for DDA. Then, the following separation gradient with a minor modification was implemented: 10%–30% buffer B from 0 to 97 min; 30%–100% buffer B from 97 to 100 min; 100% buffer B from 110 to 120 min. The same mass spectrometer was employed for DIA assay with analytic time of 2 h/sample. Each DIA cycle was composed of one full MS1 scan with a range of 350–1650 m/z (scan resolution 120,000 at 200 m/z, AGC target 3e6 and maximum IT 50 ms) and 30 MS2 scans at a DIA mode (scan resolution 30,000 at 200 m/z, AGC target 3e6, Maximum IT auto, activation type as HCD, normalized collision energy at 30 eV with spectral data type set as profile).

#### 2.1.2 Phosphoproteomic analysis

The iTRAQ‐labeled phosphopeptides were enriched and separated with a C18 column (Thermo Scientific Acclaim PepMap100, 100 μm × 2 cm, NanoViper) packed in a capillary column (Thermo Scientific EASY column, length 10 cm, ID 75 μm, particle size 3 μm, C18‐A2) and coupled to a nanoflow HPLC Easy‐nLC 1200 system (Thermo Fisher Scientific, cat#LC140) with LC gradient rate at 300 nl/min. Two buffer solutions were used, including buffer A (a mixture of formic acid:H_2_O = 1:1000 [vol/vol]) and buffer B (a mixture of formic acid:acetonitrile = 1:1000 [vol/vol]). The same mass spectrometer that was used for proteomic investigation was applied for phosphoproteomic analysis. Positive ion mode was selected for measurement. The MS1 full scan was conducted with a range of 300–1800 m/z, and with resolution of 70,000 at m/z 200, AGC target 1e6, maximum IT 50 ms, and dynamic exclusion time as 60 s. A total of 20 MS2 scans were obtained after the MS1 scan at resolution of 17,500 at 200 m/z, activation type as HCD, isolation window as 2 m/z, normalized collision energy at 30 eV, and underfill at 0.1%.

#### 2.1.3 Database searching of proteomic data

First, we used DDA mass spectrometric data to generate a DDA spectral library. DDA data analysis was conducted with MaxQuant software (version 1.5.3.17) and human UniProt database (download in September, 2019) plus iRT peptide sequence (>Biognosys|iRT‐Kit|Sequence_fusionLGGNEQVTRYILAGVENSKGTFIIDPGGVIRGTFIIDPAAVIRGAGSSEPVTGLDAKTPVISGGPYEYRVEATFGVDESNAKTPVITGAPYEYRDGLDAASYYAPVRADVTPADFSEWSKLLQFGAQGSPFLK). Of note, the DDA analysis was performed with the following parameters: trypsin as the enzyme, 2 for max missed cleavages, carbamidomethyl (C) as the fixed modification, oxidation (M), and acetyl (Protein N‐term) as the dynamic modification. Peptides and proteins were identified with a false discovery rate (FDR) <1%. Finally, spectral library was established with the Spectronaut software (Spectronaut Pulsar X_12.0.20491.4, Biognosys) by combining DDA raw files and the results of database searching.

DIA mass spectrometric data were analyzed with Spectronaut software (Spectronaut Pulsar X_12.0.20491.4, Biognosys) and referenced to above established DDA spectral library. The following parameters were used for analysis: “dynamic iRT” was selected for retention time prediction type, “enabled” was chosen for interference on MS2 level correction, while “enabled” was used for cross‐run normalization. FDR of peptide and protein was <1%. The raw proteomic data were deposited to The National Omics Data Encyclopedia (NODE) database (https://www.biosino.org/node) at Bio‐Med Big Data Center (BMBDC) affiliated with Shanghai Institute of Nutrition and Health (SINH), Chinese Academy of Sciences (CAS), with a project ID of OEP002405.

#### 2.1.4 Database searching of phosphoproteomic data

The analysis of phosphoproteomic data was carried out with MaxQuant software (version 1.5.5.1) against the human Swiss‐Prot database (version: swissoprot_human_20422_20190522). The following parameters were used for analysis: “trypsin” as the enzyme, “2” for max missed cleavages, “6 ppm” for main search, “20 ppm” for first search, “20 ppm” for MS/MS tolerance, “carbamidomethyl (C) & iTRAQ8 plex (N‐term) & iTRAQ8 plex (K)” as the fixed modifications, “oxidation (M) & acetyl (Protein N‐term) & Phospho (STY)” as the variable modifications, “reverse” as the database pattern, and “true” for included contaminants. Peptide, protein, and site were identified with an FDR <1%. The raw phosphoproteomic data were deposited to NODE database at BMBDC affiliated with SINH, CAS, with project ID of OEP002366.

### 2.2 Metabolomic analysis

Tissue and cell culture medium metabolites were extracted as previously described.^10^, ^11^ For tissue samples, approximately 20 mg of each tissue sample was weighed and a 250‐μl pre‐chilled exaction solvent mixture of chloroform, methanol, and water (vol/vol/vol = 2:5:2) was added. Samples were homogenized for 3 min and then placed in a −20°C freezer for 20 min to precipitate proteins and extract metabolites. For culture media of ESCC cell line KYSE150 (Stem Cell Bank, Chinese Academy of Sciences), 20 μl medium of each case was collected and the metabolites were extracted with the addition of pre‐chilled solvent mixture of chloroform, methanol, and water (vol/vol/vol = 2:5:2).

For above mixtures containing extracted metabolites, after centrifuging at 12,000 × *g* and 4°C for 10 min, a volume of 150‐μl supernatant was acquired and moved to a clean sample vial. Internal standards were added into the metabolite solution and the mixture was then vacuum‐dried at −20°C. The residue was derivatized by use of a two‐step procedure and then analyzed based on those previously described protocols^12^, ^13^ using the Pegasus High‐Throughput Gas Chromatography with Time‐of‐Flight Mass Spectrometer system (Leco Corporation). In brief, a 1‐μl derivatized sample was injected using a mode of splitless under temperature of 270°C for the injector. A flow rate of 1.0 ml/min was used for controlling the carrier gas helium. The temperature of the oven was set at 70°C for 2 min, then raised to 180°C (10°C/min as the increasing rate), and to 230°C (6°C/min as the increasing rate), finally to 295°C (40°C/min as the increasing rate). Oven temperature at 295°C was sustained for 5 min. Notably, the transferline interface and ion source were manipulated at temperatures of 270°C and 220°C, respectively. Mass spectrometer scan was implemented in a range of 50–550 m/z and the data were acquired at a rate of 20 spectra/s. The identities of metabolites were determined by searching the internal library constructed by chemical standards. The raw metabolomic data were deposited to NODE database at BMBDC affiliated with SINH, CAS, with a project ID of OEP002347.

### 2.3 Proteome and phosphoproteome data analysis

#### 2.3.1 Missing value imputation

In the proteomic study, there were 6507 unique proteins to be identified across 48 tissue samples. Proteins that simultaneously possessed 50% missing values in ESCC and NAT tissues were excluded. For the remaining 5511 proteins, missing values were replaced by the smallest non‐missing value in the data as previously reported.^14^

In the phosphoproteomic study, there were 3215 phosphorylated proteins along with 11,232 phosphosites to be identified. To ensure data reliability in this small sample size (*n* = 6), we extracted 2740 phosphorylated proteins along with 7186 phosphosites with no missing values for further analysis.

#### 2.3.2 Differential expression analysis

For the proteomic data, the nonparametric and paired two‐class Wilcoxon rank‐sum test with Bonferroni correction was used. FDR *q*‐values were computed using an R package qvalue (v3.10) (http://github.com/jdstorey/qvalue). Fold change (FC) values were acquired by dividing the median value of each protein in NAT samples by the median value of corresponding protein in ESCC tumors. Differentially expressed proteins (DEPs) between ESCC tumor and NAT samples were determined by Bonferroni‐adjusted *p* < .05, FDR *q* < .05, and FC cutoff as 1.5.

For the phosphoproteomic data, the paired two‐class Student's *t*‐test with Bonferroni correction was used. FC values were acquired by dividing the mean value of each protein in NAT samples by the mean value of corresponding protein in ESCC tumors. Differentially expressed phosphosites between ESCC tumor and NAT samples were determined by Bonferroni‐adjusted *p* < .05 and FC cutoff as 1.5.

### 2.4 Metabolomic data analysis

#### 2.4.1 Missing value imputation

Metabolites that simultaneously possessed 50% missing values in ESCC and NAT tissues were excluded. Subsequently, there were 200 unique metabolites to be identified across 48 tissue samples. Missing values were imputed using the random forest method as previously reported.^15^

#### 2.4.2 Differential expression analysis

We implemented nonparametric and paired two‐class Wilcoxon rank‐sum test with Bonferroni correction to identify differential metabolites. We then calculated the FDR *q*‐value for each metabolite using an R package qvalue (v3.10) (http://github.com/jdstorey/qvalue). Differential metabolites between ESCC tumor and NAT samples were determined by Bonferroni‐adjusted *p* < .05 and FDR *q* < .05.

### 2.5 Bioinformatic analysis

#### 2.5.1 GO and KEGG analyses

Gene ontology (GO) and KEGG analyses were performed using ClueGO,^16^ a software package based on cytoscape, for mRNAs and proteins. In ClueGO, pathway analysis was conducted using two‐sided hypergeometric test.

#### 2.5.2 Gene set enrichment analysis

Gene set enrichment analysis (GSEA) analysis was implemented using an R package fgsea for all proteins in this study. FC values (ESCC samples/NAT samples) of all proteins were input for computation. The enriched pathway information was pooled from the GO, Reactome, and KEGG databases.

#### 2.5.3 Proteomap analysis

We executed an approach, proteomaps,^17^ to visualize the composition of proteomes with a focus on protein abundances and functions. DEPs between ESCC and NAT samples were recruited, and median values of DEPs of ESCC group and NAT group were used for construction of proteomaps.

#### 2.5.4 Quantification of pathway activity

All pathway scores of the enrolled samples were inferred by gene set variation analysis (GSVA) method from the GSVA R package.^18^ The gene sets used for computation included KEGG, Reactome, and BIOCARTA. The Benjamini–Hochberg method was used to adjust *p*‐values of pathways between ESCC and NAT samples.

#### 2.5.5 Metabolite set enrichment analysis

Metabolite set enrichment analysis (MSEA) was carried out by using differentially expressed metabolites between ESCC tumors and NATs with an online tool MetaboAnalyst 4.0.^19^, ^20^ Before running the analysis, data of metabolites was log_2_‐transformed. During the analysis, the metabolite set library was pathway‐associated with metabolite sets (KEGG) (Oct2019).

#### 2.5.6 Identification of tumor antigens, potential cancer drivers and kinases

Proteomic data were used for identification of tumor antigens, potential cancer drivers and kinases. A cancer/testis (CT) antigen list was downloaded from the CTDatabase (http://www.cta.lncc.br/modelo.php). A potential cancer driver list was acquired from a previous study.^21^ A kinase list was obtained from the databases of PhosphoSitePlus and NetworKIN.^22^ By comparison to these lists, tumor antigens, potential cancer drivers and kinases were identified.

#### 2.5.7 Drug annotation of potential cancer drivers and key kinases

In this study, two drug databases, DrugBank^23^ and PKIDB,^24^ were used to annotate those identified potential cancer drivers and key kinases with available drugs or inhibitors.

## 3 RESULTS

### 3.1 Molecular landscape of ESCC by multilayer omics profiling

**FIGURE 1:**
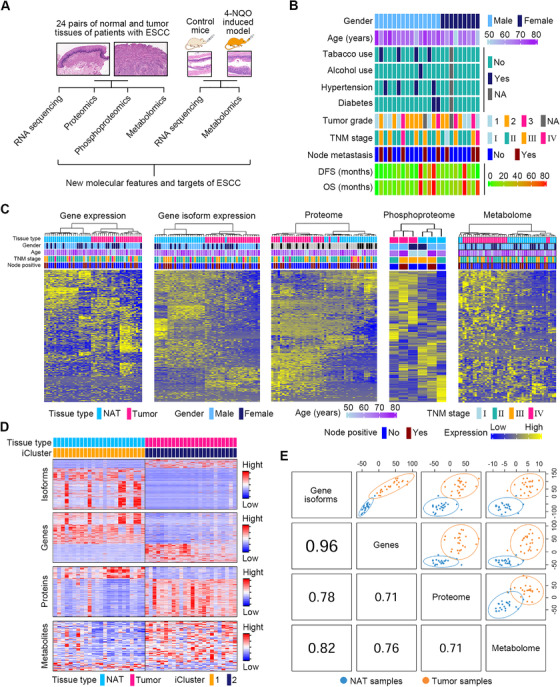
Multilayer omics profiling of ESCC and NAT tissues. (A) Strategy of multilayer omics investigations using paired ESCC and NAT samples from patient cohort 1 (*n* = 24) along with esophageal tissues from control mice and carcinogen‐induced ESCC mice. (B) Heatmap showing clinical parameters of ESCC patients for multilayer omics investigations. (C) Unsupervised hierarchical clustering of multi‐omics data of ESCC patients. Clinical parameters from 48 tissue samples profiled with each omics data are also depicted. (D) Integrated clustering of four molecular layers of data showed that tissue samples of patients with ESCC fell into two groups by iCluster that were virtually identical to histological classes ESCC and NAT. (E) Correlation analysis between any two omics profiles using DIABLO algorithm

We enrolled 24 ESCC patients (treatment‐naive cases) as cohort 1 and harvested their paired tumor and NAT samples. Each sample underwent RNA‐sequencing (RNA‐seq), data‐independent acquisition (DIA)‐based proteomic and nontargeted metabolomic investigations (Figure 1A). Due to the limitation of tissue samples, only three pairs of tumor and NAT tissues were selected for phosphoproteomic assay using mass spectrometry‐based isotope tagging for relative and absolute quantification (iTRAQ). Furthermore, esophageal tissues from control mice and ESCC mice induced by the carcinogen 4‐nitroquinoline‐1‐oxide (4‐NQO) were collected for RNA‐seq and nontargeted metabolomic surveys in order to verify the conservation of ESCC molecular features between distinct species (Figure 1A). Clinical characteristics of ESCC patient cohort 1 are summarized in Figure 1B.

The quality of samples and data was stringently controlled. For patients, their ESCC regions with >80% tumor cells (median [range]: 90% [80%–98%]) were harvested for analysis. The sample quality of RNAs and proteins from all specimens was verified (Figure S1, Table S1). For RNA‐seq samples, the sequencing library quality was determined by Agilent 2100 Bioanalyzer, and data quality was evaluated by the Phred quality score (Table S2, Figure S2A). For proteomics analyses, the variability of quality control (QC) samples, data points for each peak, peak capability, internal calibration standards, and distribution of protein false discovery rate (FDR) were analyzed, indicating negligible instrument drift and high quality of the data (Figure S2B–F). For phosphoproteomics analyses, mass error distribution, phosphorylated peptide score distribution, and phosphorylated peptide ratio distribution were assessed (Figure S2G–I), indicating data of high quality. For metabolomics profiling, low variability of QC samples was observed, indicating stability of the measurement system (Figure S2J). For mice, the pathophysiological characteristics of their esophageal tissues were confirmed using hematoxylin and eosin (H&E) staining together with immunohistochemistry (IHC) staining for the esophageal marker keratin 14 and cell proliferation marker Ki‐67 (Figure S3A). Furthermore, RNA‐seq data quality was confirmed by the Phred score (Figure S3B).

To confirm whether our data were able to accurately capture the molecular features of human ESCC, we first examined the expression of well‐established ESCC markers between ESCC tumors and NATs of patients using our multi‐omics data. The esophageal carcinogenesis marker keratin 14 (K14, encoded by *KRT14*), growth factor receptors epidermal growth factor receptor (EGFR, encoded by *EGFR*), epidermal growth factor receptor 2 (HER2, encoded by *ERBB2*), nuclear receptor cyclin D1 (encoded by *CCND1*), cell proliferation markers proliferating cell nuclear antigen (PCNA, encoded by *PCNA*), and Ki‐67 (encoded by *MKI67*) were selected for analysis. As reported previously, cyclin D1 expression is a common genetic alteration as well as a key driver of ESCC.^25^ Furthermore, K14, EGFR, cyclin D1, PCNA, and Ki‐67 are upregulated both at RNA and protein levels, while HER2 is elevated only at the protein level in ESCC tissues.^26^, ^27^, ^28^, ^29^, ^30^, ^31^ In agreement with these previous findings, our RNA‐seq data demonstrated that *KRT14*, *EGFR*, *CCND1*, *PCNA*, and *MKI67*, but not *ERBB2*, were transcriptionally upregulated in ESCC tissues (Figure S4A). Additionally, our proteomic data revealed that K14, EGFR, HER2, PCNA, and Ki‐67 were all increased in ESCC tissues (Figure S4B). However, cyclin D1 was not identified in our proteomic investigation. Therefore, the results of our multi‐omics data identified many of the well‐known molecular features of ESCC.

Prior to thoroughly analyzing the molecular features of ESCC using our multi‐omics data, it was important to ascertain whether our omics data were consistent with established omics datasets. A previous study enrolled 53 ESCC patients, and collected their tumor tissues and matched NAT tissues for gene expression profiling.^28^ This study revealed that 116 genes were dramatically upregulated, while 43 genes were strikingly downregulated in ESCC tumor tissues. In our RNA‐seq data of ESCC patients, among those previously reported, upregulated, 116 genes, 76 of them (65.52%) were remarkably increased in ESCC tumor tissues (Figure S5A). In addition, 32 out of the previously 43 reported downregulated genes (74.41%) were found to be downregulated in the ESCC tumor tissues of our patient cohort (Figure S5B). This result indicated the consistency between our omics data and the published omics data.

Alternative splicing (AS) of mRNA allows for the expression of multiple RNA isoforms and contributes to the complexity of the proteome.^32^, ^33^ Hence, we examined gene expression including their respective isoforms from the RNA‐seq data. We carried out unsupervised hierarchical clustering and principal component analysis (PCA) using the multilayer omics data to ascertain human ESCC molecular features. When compared to NATs, ESCC tissues showed distinct signatures at gene, gene isoform, protein, phosphoprotein, and metabolite levels (Figures 1C and S6). As the phosphoproteomic investigation was only executed in three pairs of samples, phosphoproteomic data were excluded in the subsequent multivariate analysis. Next, integrated clustering of RNA‐seq, proteomic and metabolomic data was performed using the iCluster algorithm,^34^ and the results clearly discriminated ESCC from NAT samples (Figure 1D). Finally, we executed the DIABLO algorithm^35^ to extract molecular profiles from the omics data for all patient samples, which allowed computation of the correlation between any two molecular profiles. The result revealed strong correlations between any two omics profiles (Figure 1E), demonstrating a consistent difference between ESCC and NAT samples at distinct molecular layers.

Subsequently, the RNA‐seq and metabolomic data of mice were used to execute unsupervised hierarchical clustering and PCA analysis. As compared to esophageal tissues from control mice, esophageal tissues from ESCC mice exhibited distinct molecular features at gene, gene isoform, and metabolite levels (Figure S7), demonstrating that the alteration of molecular features in ESCC was conserved between different species.

### 3.2 Discordance between transcriptome and proteome revealed active posttranscriptional and posttranslational regulation

To ascertain the global association between transcriptome and proteome, we computed gene‐wise (inter‐sample) correlation of 6174 mRNA–protein pairs across all patient samples. We observed a striking discordance between mRNAs and proteins as showed by a median gene‐wise correlation value of 0.07 and a low percentage (15.69%) of mRNA–protein pairs with significant positive Spearman correlations (Figure S8A). Discordance between mRNAs and protein expression levels indicated active posttranscriptional regulation in these tissue samples.

**FIGURE 2:**
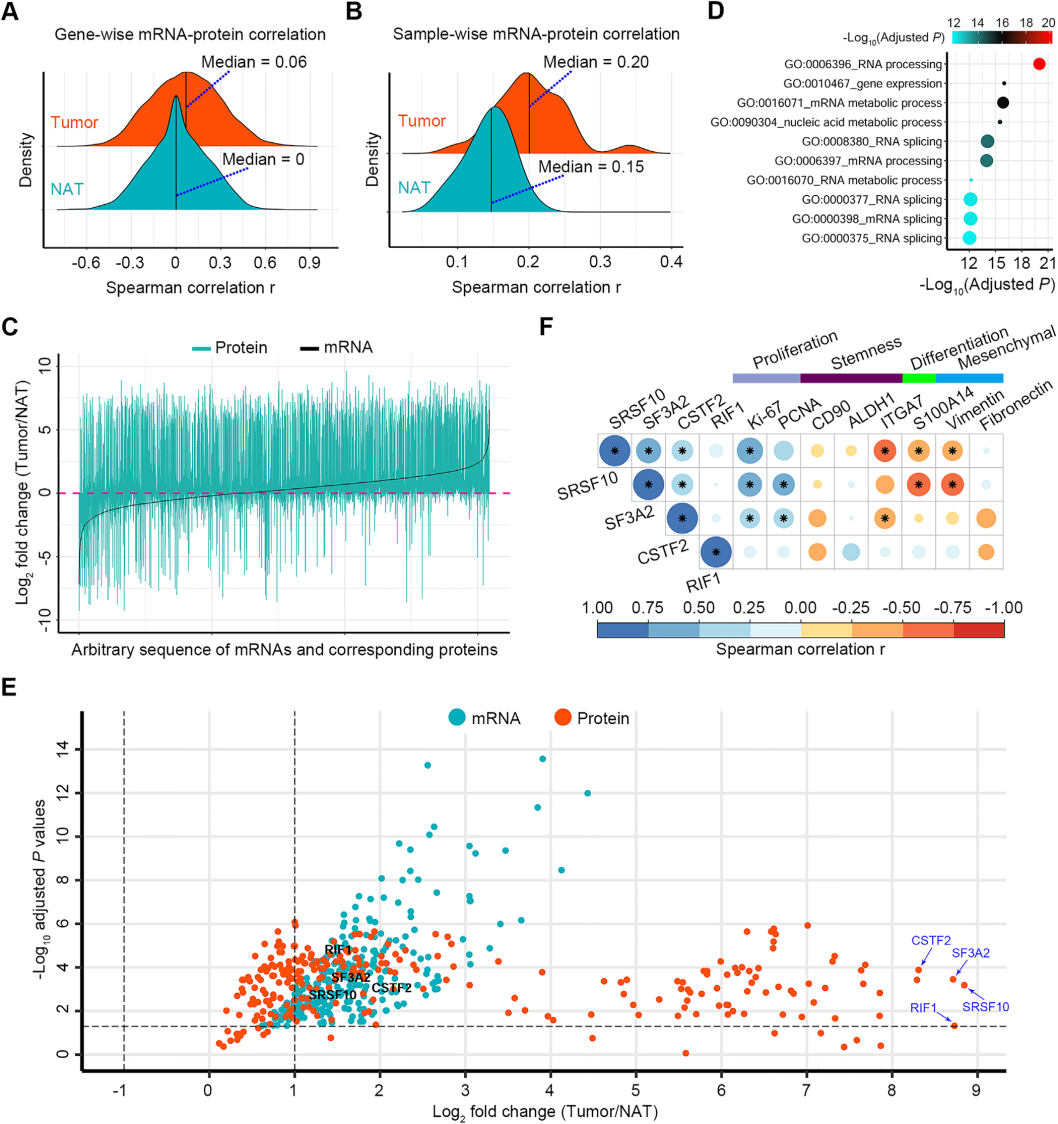
Correlations between transcriptomic and proteomic data of ESCC patients. (A and B) Density ridgeline plots showing gene‐wise correlations (A) and sample‐wise correlations (B) in tumors and NATs, respectively. (C) Multiple line plots displaying the fold change (FC) values of mRNAs and corresponding proteins between ESCC tumors and NATs. mRNAs were aligned by their FC values with an increasing order. (D) GO biological process analysis of 480 genes with high transcriptional and translational activities in ESCC tumors. The top 10 enriched pathways were selected for presentation. Each node size represents the percentage of measured genes in a specific pathway. (E) Volcano plot displaying the FC values (log_2_ transformed) and *p*‐values (‐log_10_ transformed) of 480 genes with high transcriptional and translational activities in ESCC tumors. *p*‐Values of RNAs were derived from edgeR analysis by comparing ESCC and NAT samples, while *p*‐values of proteins were obtained from the nonparametric and paired two‐class Wilcoxon rank‐sum test with Bonferroni correction by comparing ESCC and NAT samples. (F) Spearman correlation analysis between four proteins with extremely high expression in ESCC tumors and key neoplastic markers. Star marks in heatmap cells represent the correlations between proteins of interest and neoplastic markers with *p* < .05. The areas of circles show the absolute value of corresponding correlation coefficients

The next step involved determination of the mRNA–protein relationship differences between the ESCC and NAT samples of patients. First, we executed gene‐wise correlation analysis. Although a remarkable discrepancy between mRNAs and protein expression levels was observed in both groups of samples as shown by the low median correlation of 0.06 for ESCC tissues and 0 for NATs, the tumor tissues exhibited a right shift of distribution of correlation values relative to NATs (Kolmogorov–Smirnov test *p *< 2.2 × 10^–16^) (Figure 2A), indicating more positive correlations in tumor tissues. Next, we calculated sample‐wise (intra‐sample) correlations between mRNAs and proteins. The median correlation values for ESCC and NAT tissues were 0.20 and 0.15, respectively. Notably, ESCC tissues displayed an overt right shift of distribution of correlation values as compared to NATs (Kolmogorov–Smirnov test *p* = 2.34 × 10^–5^) (Figure 2B), demonstrating more positive correlations in tumor tissues. Furthermore, analysis of the FC of mRNA and protein expression between ESCC tumors and NATs showed that most of proteins were upregulated in tumor tissues not only when corresponding mRNAs were increased, but also when corresponding mRNAs were undisturbed or reduced (Figure 2C). However, most of proteins were downregulated in NAT tissues regardless of any expression patterns of corresponding mRNAs (Figures 2C and S8B). Additionally, more upregulated mRNAs were observed in ESCC tumors relative to NATs (Figure 2C). Together, these results indicated that mRNA transcription and translation were enhanced, and posttranscriptional regulation was hyperactive in ESCC tissues. Moreover, to verify the active posttranscriptional control in ESCC, we performed validation assay using three randomly selected genes with negative correlation between their mRNAs and proteins, including *SMNDC1*, *MTHFD2*, and *PNO1*. RT‐qPCR and Western blotting measurements showed that although mRNAs of these genes were not altered in ESCC tissues as relative to NATs, their protein products SMNDC1, MTHFD2, and PNO1 were markedly elevated (Figure S8C,D), thus underscoring the high activity of posttranscriptional regulation in ESCC.

We hypothesized that those genes with increased expression at both mRNA and protein levels (namely with high transcriptional and translational activities) in ESCC tumors acted as core upstream signals that led to the observed difference in posttranscriptional modulation between ESCC and NAT tissues. To validate our hypothesis, we enrolled genes (*n* = 480, accounting for 7.77% [480/6174]) with increased mRNA abundance in tumor tissues and with positive correlation between mRNAs and corresponding proteins across all tissues for analysis. GO biological process analysis revealed that activities of RNA processing, RNA slicing, and gene expression were remarkably enhanced in ESCC tumors as evidenced by the top 10 enriched pathways (Figures 2D and S8E). Of importance, among these 480 genes, those producing proteins with extremely high levels in ESCC tissues, included serine and arginine rich splicing factor 10 (SRSF10), splicing factor 3A subunit 2 (SF3A2), cleavage stimulation factor subunit 2 (CSTF2), and replication timing regulatory factor 1 (RIF1) (Figure 2E). In our proteomic data for ESCC tumors, SRSF10, SF3A2, and CSTF2 were positively correlated to proliferation markers Ki‐67 and/or PCNA (Figure 2F). In addition, SRSF10 and SF3A2 were negatively associated with differentiation marker S100A14 (Figure 2F). These results indicated that SRSF10, SF3A2, and CSTF2 may actively participate in ESCC malignancy.

**FIGURE 3:**
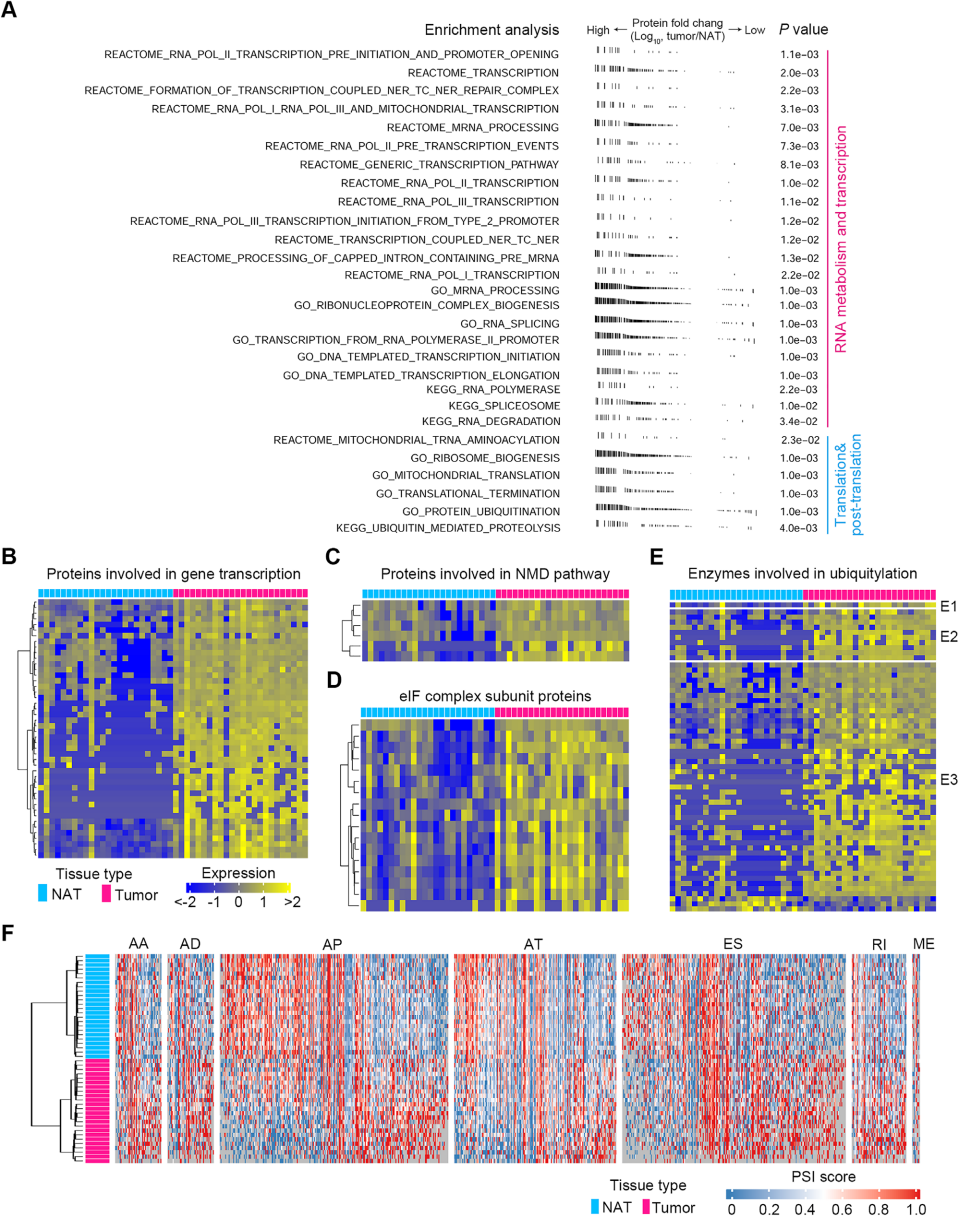
Mechanistic investigations of transcriptional, posttranscriptional, translational, and posttranslational modulations in ESCC. (A) GSEA analysis of all proteins showing active gene sets involved in transcriptional, posttranscriptional, translational, and posttranslational level control in human ESCC tissues. One vertical line in the figure represents one protein. (B–E) Differentially expressed proteins between ESCC and NAT samples involved in gene transcription (B), NMD pathway (C), eIF complex subunit (D), and ubiquitylation (E). (F) Heatmap displaying RNA transcripts with statistically differential PSI values between ESCC and NAT samples. Seven alternative splicing events were enrolled for analysis

We then hypothesized that the protein activities described above were involved in upstream signaling that would affect posttranscriptional control via altering the proteome. Hence, we performed GSEA using all proteins and selected gene sets involved in posttranscriptional and posttranslational level control (*p* < .05). As expected, tumors were significantly more enriched than NATs in transcription and translation‐related functions, including RNA processing/transcription, protein translation, protein modification, and proteolysis (Figure 3A). Then, we extracted proteins key for posttranscriptional and posttranslational regulation, including members of RNA transcription, the nonsense‐mediated mRNA decay (NMD) pathway that regulates the abundance of a large number of cellular RNAs,^36^ eukaryotic initiation factor (eIF) complex key for protein translation, and protein ubiquitin proteasome system (UPS) key for protein degradation. Among them, most of the proteins with differential expression between ESCC and NAT tissues showed increased upregulation in tumors (Figure 3B–E), consistent with the expedited rates of RNA transcription, RNA decay, protein translation, and proteolysis in ESCC tissues. Among those DEPs between ESCC and NAT samples involved in pathways listed in Figure 3A, five of them with extremely high expression in ESCC are highlighted in Figure S9, including SRSF10, U6 snRNA‐associated Sm‐like protein LSM6, SF3A2, mitochondrial ribosomal protein L21 (MRPL21), and ubiquitin conjugating enzyme E2 A (UBE2A).

It is known that pre‐mRNA alternative splicing (AS) is a key posttranscriptional process.^37^ To provide more evidence for the active posttranscriptional regulation in ESCC, we analyzed seven common AS events in our RNA‐seq data, including alternate acceptor site (AA), alternate donor site (AD), alternate promoter (AP), alternate terminator (AT), exon skip (ES), mutually exclusive exons (ME), and retained intron (RI) using the SpliceSeq tool.^38^ We calculated percent‐splice‐in (PSI) values of RNA transcripts, which reveal how efficiently these sequences are spliced into transcripts for a specific AS event.^39^ There were 3883 RNA transcripts with statistically differential PSI values between ESCC tumors and NATs, and more AA, AD, AT, ES, and RI events were observed in tumors (Figure 3F). This was consistent with previous studies that showed increased AS events in breast cancer and colorectal cancer tissues relative to NAT tissues.^40^, ^41^

### 3.3 Integrative illustration of molecular pathways and metabolic signatures

**FIGURE 4:**
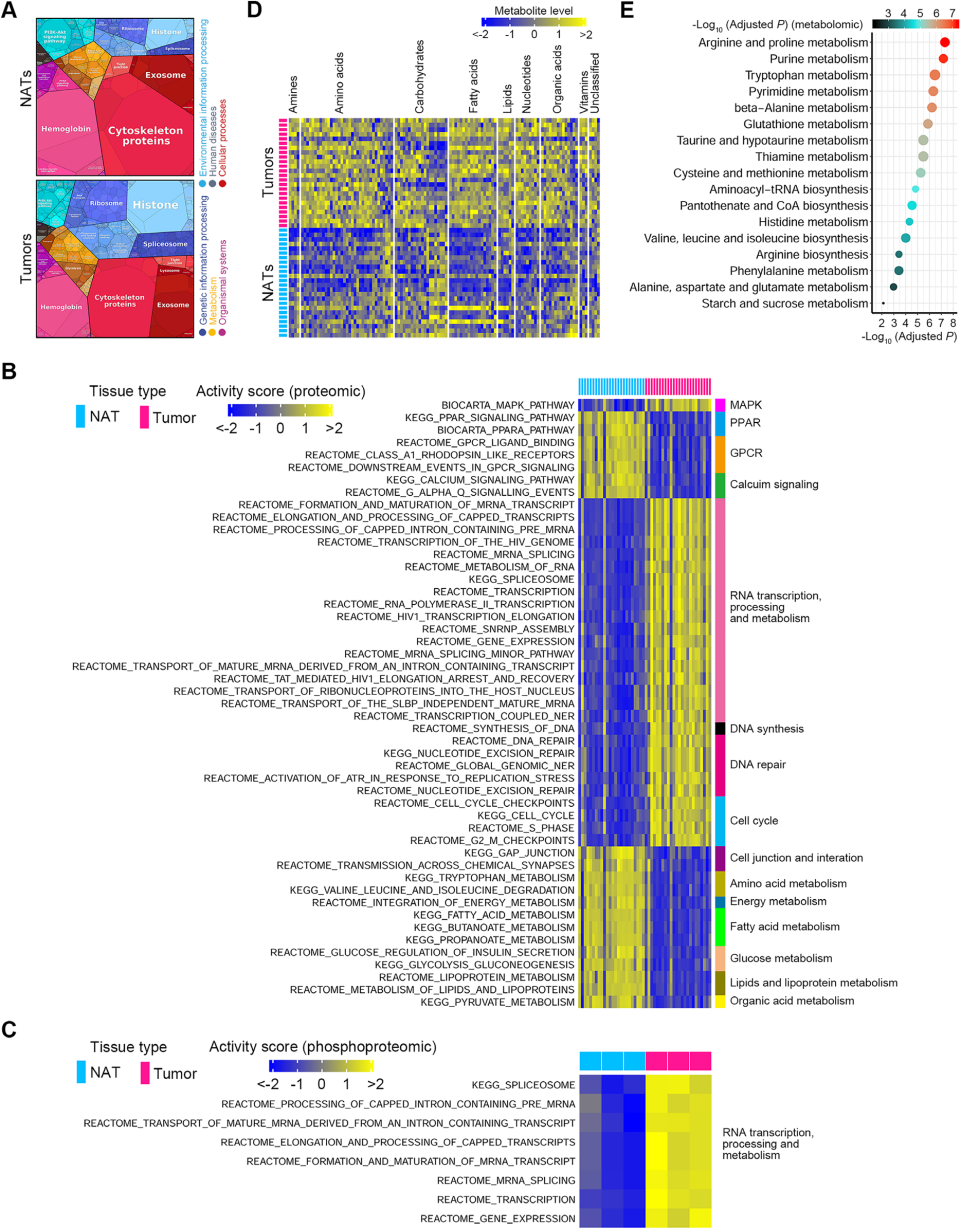
Multi‐omics characterization of molecular features of ESCC. (A) Differential functional categories between NAT and ESCC specimens as illustrated by Proteomaps using proteomic data. Each polygon corresponds to a single KEGG pathway, and the size was correlated with the ratio between the two groups of samples. The maps show high dissimilarity between NAT and ESCC tissues. (B) Activity scores of the top 50 significantly perturbed pathways according to protein levels between ESCC and NAT tissues. Up‐ and downregulated pathways are shown as blue and yellow, respectively. (C) Activity scores of significantly disturbed pathways according to protein phosphorylation levels between ESCC and NAT tissues. Up‐ and downregulated pathways are indicated as blue and yellow, respectively. (D) Differentially expressed metabolites between ESCC tumors and NATs as exhibited by the heatmap. Notably, most of these metabolites were upregulated in ESCC tumors. (E) Significantly perturbed metabolic pathways in human ESCC. The node size represents the statistic *q*‐values of metabolic pathways derived from MSEA analysis

Due to the high discordance between mRNAs and proteins, we used proteomic data together with phosphoproteomic and metabolomic data to delineate the multilayer molecular alterations of human ESCC. First, we analyzed pathways dysregulated in ESCC using proteomic data. There were 2890 DEPs in ESCC tissues (adjusted *p* < .05, FDR *q* < .05, FC cutoff as 1.5) (Figure S10A). We then constructed proteomaps^17^ to cluster the DEPs according to their KEGG pathway annotations and observed a distinct difference between ESCC tumors and NATs (Figure 4A). ESCC tumors were dominated by higher levels of spliceosome, histone, and ribosome‐related proteins along with lower levels of cytoskeleton proteins. Subsequently, we performed GSVA^18^ using those DEPs to quantify pathway activation. A total of 157 pathways were found to be significantly perturbed in ESCC tumors (adjusted *p* < .05) (Figures 4B and S11). Predominantly activated pathways in ESCC included those related to RNA transcription/processing/metabolism, DNA synthesis and repair, protein synthesis, proteolysis, and cell cycle. Conversely, pathways related to cell junctions and interactions were strikingly attenuated in ESCC tumors. Additionally, ectopic stimulation of oncogenic signaling pathways, including MAPK, Notch, Wnt, and mTOR, was found in ESCC tumors. We then extended pathway analysis to the phosphoproteomic data. There were 517 differentially expressed phosphosites in ESCC tumors (adjusted *p* < .05, FC cutoff as 1.5) (Figure S10B), indicating increased activity of posttranslational modification. GSVA analysis using the corresponding proteins of these phosphosites revealed the activation of pathways involved in RNA transcription, processing, and metabolism in human ESCC tumors (adjusted *p* < .05) (Figure 4C).

Subsequently, we analyzed the metabolic signatures of human ESCC using metabolomic data. There were 56.50% metabolites (113/200) with differential expression in ESCC tumors (adjusted *p* < .05, FDR *q* < .05). Among these differentially expressed metabolites (DEMs), 83.19% (94/113) of them were upregulated (Figure 4D), indicating an active metabolic feature of ESCC tumors. We then performed MSEA^19^, ^20^ using those 113 DEMs and observed a total of 17 metabolic pathways with remarkable perturbation in ESCC tumors (adjusted *p* < .05, FDR *q* < .05) (Figure 4E). Of note, 58.82% (10/17) of these pathways were involved in amino acid metabolism, indicating that amino acid metabolism was predominantly disturbed in human ESCC. In line with this finding, measurement of spent culture media of a human ESCC cell line KYSE150 manifested that ESCC cells readily imported and consumed many extracellular amino acids, including cystine, arginine, proline, and so on (Figure S12).

**FIGURE 5:**
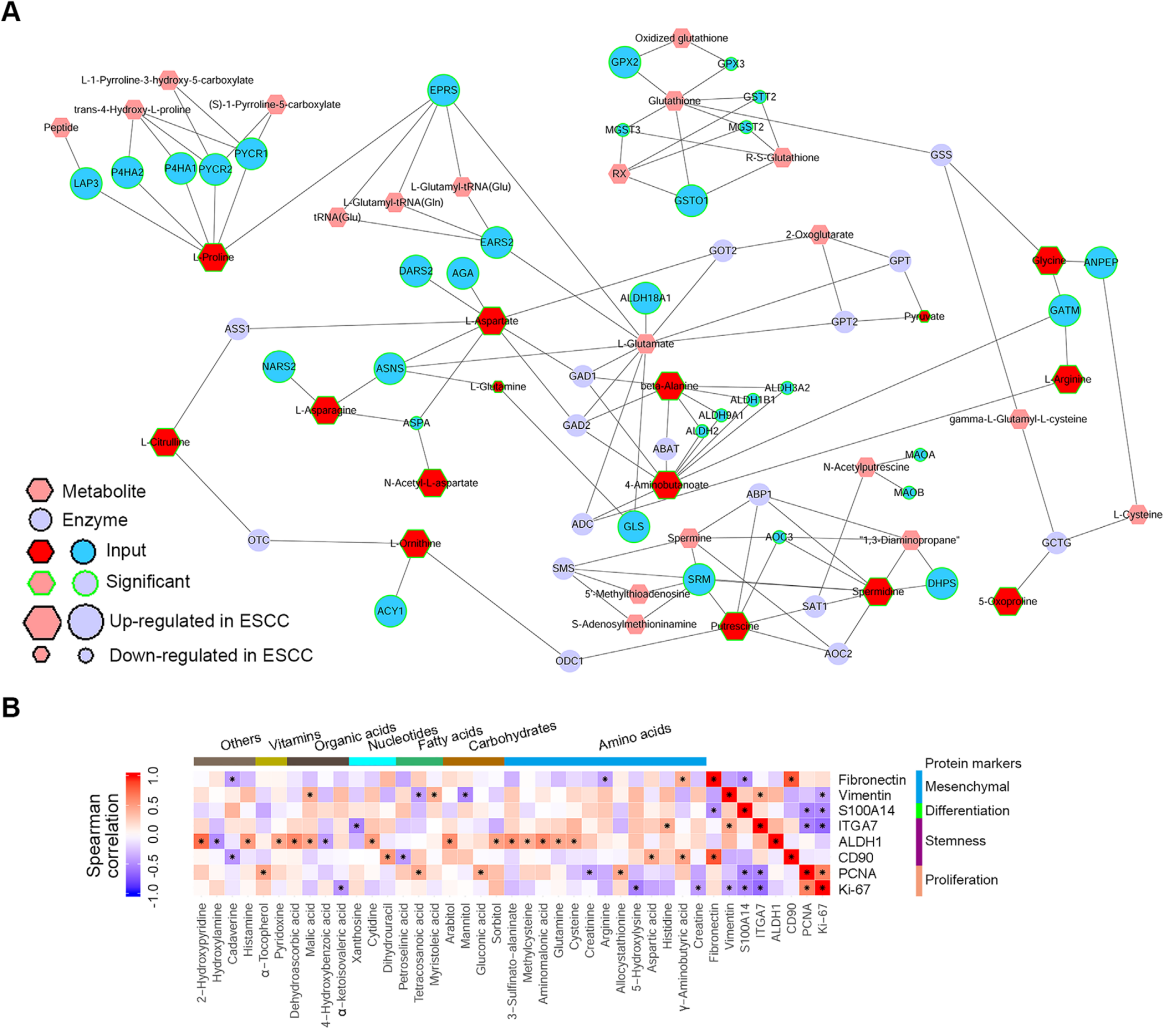
Integrative analysis of molecular signatures of ESCC using proteomic and metabolomic data. (A) Integrative analysis of significantly altered metabolic enzymes and metabolites for the most perturbed metabolic pathway in ESCC, arginine and proline metabolism. (B) Spearman correlation analysis between DEMs and DEPs representing key cellular phenotypes of ESCC. Metabolites with significant correlation to at least one phenotypic protein marker are selected for display. Star marks in heatmap cells represent correlations between metabolites and proteins with *p* < .05

Both proteomic and phosphoproteomic data pinpointed an increased activity in pathways related to RNA transcription, processing, and metabolism, indicating that these pathways were crucial for ESCC pathobiology. These pathways are thoroughly analyzed in the next section. We then performed an integrated analysis for the most perturbed metabolic pathway in ESCC, arginine and proline metabolism, using metabolomic and proteomic data. The network highlighted that ESCC tumors selectively expressed several metabolic enzymes to expedite amino acid production (Figure 5A).

In view of the importance of metabolites in cellular phenotype determination,^42^, ^43^ we conducted correlation analysis between DEMs and DEPs representing key cellular phenotypes of ESCC (Figure 5B). Overtly, 13 metabolites showed positive linkage to aldehyde dehydrogenase 1 (ALDH1), a well‐established stemness marker of ESCC.^44^, ^45^ These metabolites included five amino acids (cysteine, glutamine, aminomalonic acid, methylcysteine, and 3‐sulfinato‐alaninate), two carbohydrates (sorbitol and arabitol), one nucleotide (cytidine), two organic acids (malic acid and dehydroascorbic acid), one vitamin (pyridoxine), and two unclassified metabolites (histamine and 2‐hydroxypyridine). These results suggested that altered metabolites might be involved in impacting ESCC stemness.

### 3.4 Stimulated pathways of RNA transcription, processing, and metabolism unraveled by proteomic and phosphoproteomic profiling

**FIGURE 6:**
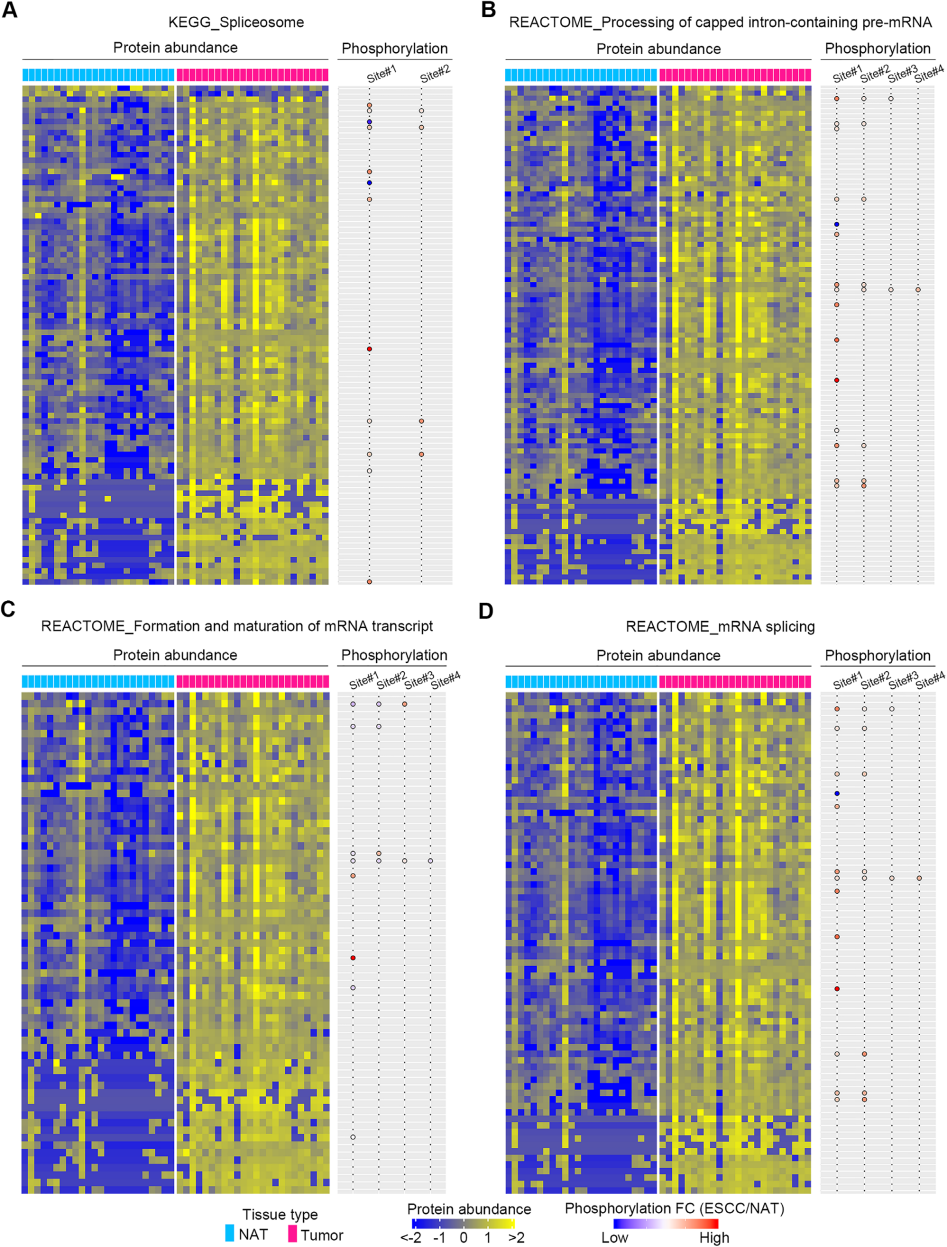
Essential pathways of ESCC identified by the integrative analysis of proteomic and phosphoproteomic data. (A–D) Heatmaps showing DEPs between ESCC tumors and NATs in the enriched pathways, including spliceosome (KEGG), processing of capped intron‐containing pre‐mRNA (REACTOME), formation and maturation of mRNA transcript (REACTOME), and mRNA splicing (REACTOME). Balloon plots on the right reveal differential phosphosites of proteins in the matched heatmaps on the left

As mentioned above, both proteomic and phosphoproteomic data pinpointed those pathways involved in RNA transcription, processing, and metabolism (Figure 4B,C), thus implicating the importance of these pathways for ESCC malignancy. Consequently, the features of these pathways were thoroughly dissected. The percentage values of remarkably upregulated proteins in ESCC tumors accounting for totally enriched and differential proteins in each pathway were 97.75% (87/89), 100.00% (99/99), 100.00% (67/67), 100.00% (77/77), 100.00% (52/52), 100.00% (62/62), and 100.00% (63/63) for spliceosome (KEGG), processing of capped intron‐containing pre‐mRNA (REACTOME), formation and maturation of mRNA transcript (REACTOME), mRNA splicing (REACTOME), transport of mature mRNA derived from an intron‐containing transcript (REACTOME), elongation and processing of capped transcripts (REACTOME), and transcription (REACTOME), respectively (Figures 6 and S13). While the percentage values of significantly enhanced phosphosites in ESCC tumors accounting for totally enriched and differential phosphosites in each pathway were 87.50% (14/16), 96.15% (25/26), 100.00% (15/15), 95.83% (23/24), 95.00% (19/20), 93.75% (15/16), and 100.00% (17/17), respectively (Figures 6 and S13). Collectively, these findings showed that these molecular pathways were aberrantly stimulated in ESCC tumors.

### 3.5 Identification of protein markers with prognostic potential

**FIGURE 7:**
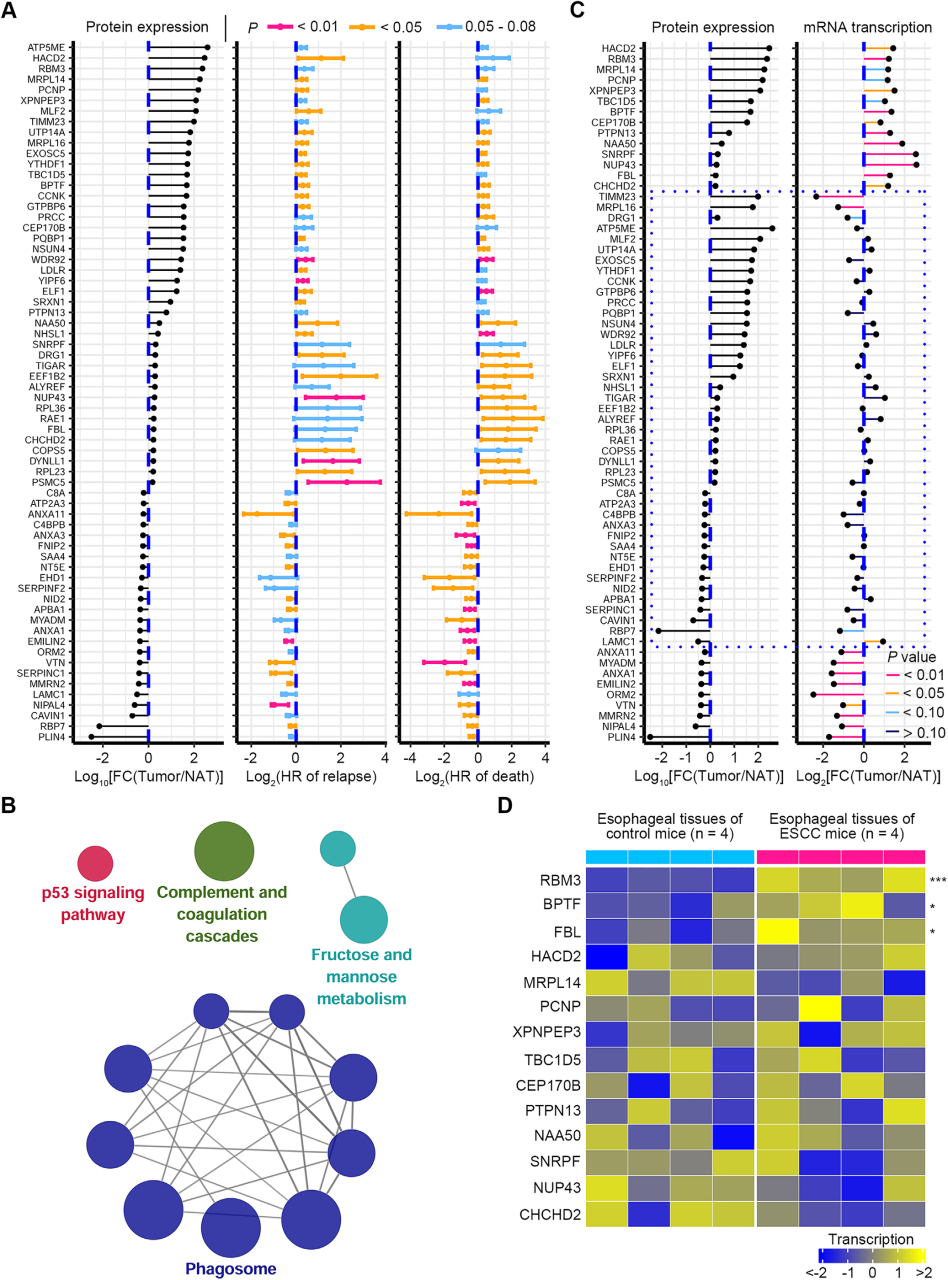
Discovery of protein markers with prognostic value. (A) Total of 66 DEPs with positive correlation to HRs of disease relapse and death were identified. For these prognostic proteins, their 95% confidential intervals of HRs of relapse or death are illustrated. HRs >1 correspond to an increased risk of death/relapse compared with the lower abundance of proteins, while HRs <1 correspond to a reduced risk of death/relapse compared with the lower abundance of proteins. (B) KEGG pathway analysis showing that 118 proteins with prognostic potential are enriched in pathways (*p* < .05), including phagosome, complement and coagulation cascades, p53 signaling pathway, and fructose and mannose metabolism. Each node represents a pathway, with node size reflecting pathway enrichment significance. (C) Expression direction (tumor/NAT) between 66 proteins with prognostic potential and their corresponding mRNAs. Twenty‐three of these proteins that are out of the dashed quadrilateral reveal consistent expression direction with their corresponding mRNAs, whereas the remaining proteins that are highlighted by the dashed quadrilateral display inconsistent expression direction with their corresponding mRNAs. (D) For the 14 genes with increased expression at both mRNA and protein levels in human ESCC tumors shown in (C), their mRNA transcription was analyzed in esophageal tissues of ESCC and control mice. Three of these genes are significantly upregulated in esophageal tissues of ESCC mice. ^*^ *p* < .05, ^***^ *p* < .001, two‐tailed Student's *t*‐test

Of interest, for the DEPs in ESCC tissues of patient cohort 1, we sought to identify those proteins closely associated with patient survival and potentially involved in ESCC progression. In this small cohort (*n* = 24), higher TNM stage and lymph node metastasis, two well‐known risk factors for ESCC, were associated with increased hazard of disease relapse and death with a borderline significance (Figure S14A), indicating the robustness of the prognostic data. Univariate Cox model was fitted to assess the association between each protein and patient survival. In order to enhance the recognition of potential prognostic proteins in this small cohort, we selected a borderline *p*‐value of .08 together with a 95% confidence interval of hazard ratios (HRs) to perform statistical analysis as previously reported,^46^ and a total of 118 proteins with prognostic potential were identified. Among these proteins, 66 of them displayed positive associations with hazard of disease relapse or death (Figure 7A), while the remaining proteins exhibited negative linkage to hazard of disease relapse or death (Figure S14B). KEGG pathway analysis revealed that these 118 prognostic proteins were mainly enriched in pathways of phagosome, complement and coagulation cascades, p53 signaling pathway, and fructose and mannose metabolism (*p* < .05) (Figure 7B).

For the 66 prognostic proteins positively linked to HRs, their changes in ESCC tissues of patients at protein and corresponding mRNA levels were analyzed. Twenty‐three of these proteins showed consistent expression direction with their corresponding mRNAs, indicating that these proteins were modulated at the transcriptional level (Figures 7C and S14C). While the remaining prognostic proteins exhibited inconsistent expression direction with their corresponding mRNAs, indicating that these proteins were regulated at the posttranscriptional level (Figures 7C and S14C). Of note, 14 genes, including 3‐hydroxyacyl‐CoA dehydratase 2 (*HACD2*), RNA binding motif protein 3 (*RBM3*), mitochondrial ribosomal protein L14 (*MRPL14*), PEST proteolytic signal containing nuclear protein (*PCNP*), X‐prolyl aminopeptidase 3 (*XPNPEP3*), TBC1 domain family member 5 (*TBC1D5*), bromodomain PHD finger transcription factor (*BPTF*), centrosomal protein 170B (*CEP170B*), protein tyrosine phosphatase non‐receptor type 13 (*PTPN13*), N‐alpha‐acetyltransferase 50, NatE catalytic subunit (*NAA50*), small nuclear ribonucleoprotein polypeptide F (*SNRPF*), nucleoporin 43 (*NUP43*), fibrillarin (*FBL*), and coiled‐coil‐helix‐coiled‐coil‐helix domain containing 2 (*CHCHD2*), were significantly upregulated at both protein and mRNA levels (Figure 7C). The mRNA transcription of these genes was assessed in ESCC mice induced by 4‐NQO. The result showed that three of them, including *RBM3*, *BPTF*, and *FBL*, were significantly increased in esophageal tissues of ESCC mice as relative to that of control mice (Figure 7D), demonstrating that upregulation of these three genes in ESCC was conserved in distinct species. Next, we selected five of the most upregulated proteins in ESCC tumors, including HACD2, RBM3, MRPL14, PCNP, and XPNPEP3, together with BPTF and FBL for further investigation.

### 3.6 Validation and functional assays highlighting FBL as a new unfavorable prognostic biomarker

**FIGURE 8:**
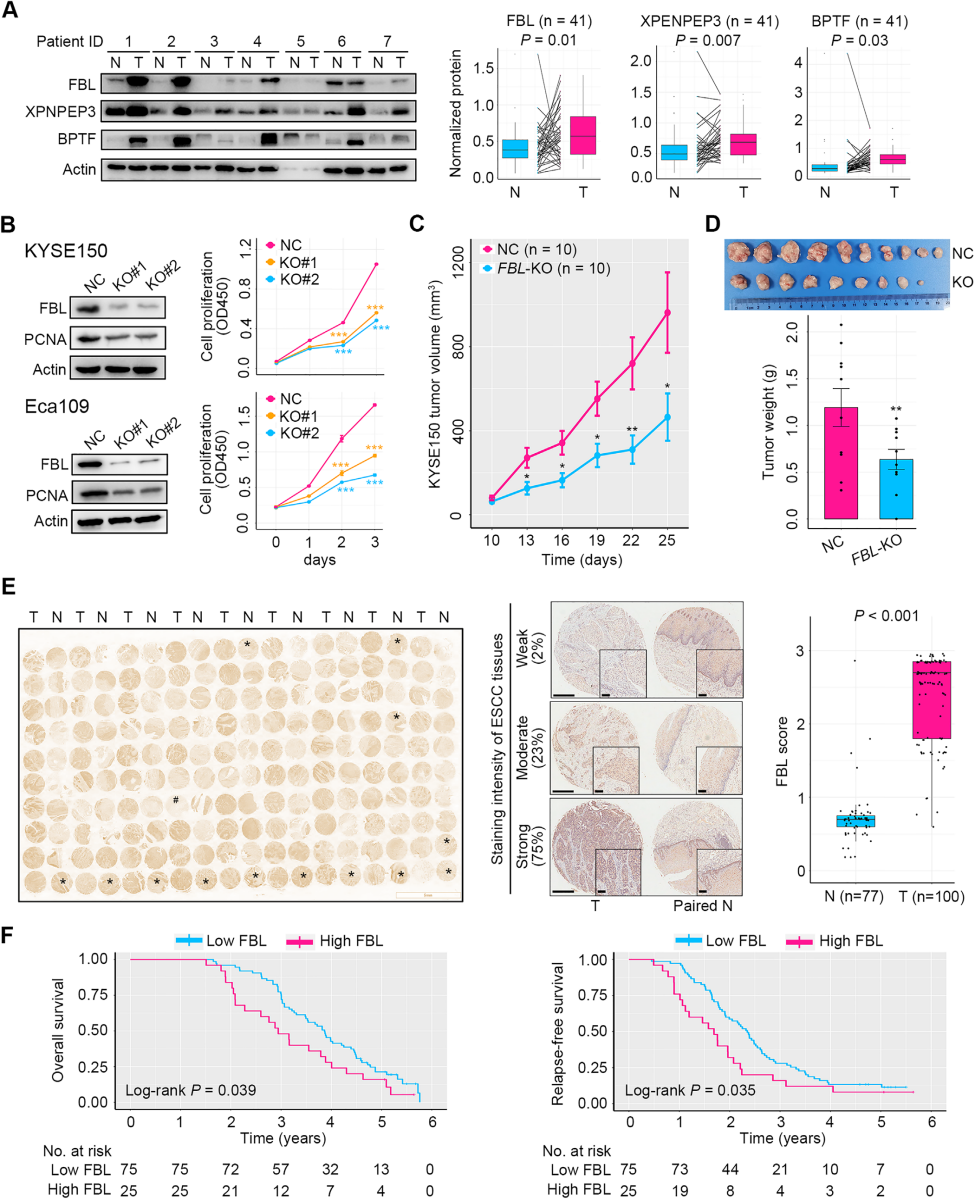
Validation and functional assays of prognostic protein markers. (A) Validation of the expression of FBL, XPNPEP3, and BPTF between ESCC tumors and NATs using a patient cohort 2 (*n* = 41). Western blot and quantitative results displayed an increase in expression of these proteins in ESCC tumors (T) relative to NATs (N). Representative Western blot images are shown. *p*‐Values were computed using the Wilcoxon rank‐sum test. (B) Knockdown of *FBL* in ESCC cells KYSE150 and Eca109 and the consequential impact on PCNA expression and cell growth in vitro. (C) *FBL* ablation curtailed the growth of KYSE150 xenograft tumors (*n* = 10 for each group). (D) Images and weight of KYSE150 xenograft tumors with or without *FBL* deletion (*n* = 10 for each group). (E) Left panel, IHC staining image showing FBL expression between ESCC (T) and NAT (N) tissues from patient cohort 3 (*n* = 100). Samples in the microtissue array that are not orderly arranged are marked with stars for ESCC tissues or hashes for NATs. Middle panel, representative images of ESCC tissues with weak, moderate, or strong FBL staining. The percentage of tumor tissues of each staining intensity is revealed. Paired normal tissues are displayed. Right panel, quantification of IHC staining. *p*‐Value was obtained from the Wilcoxon rank‐sum test. (F) Overall survival and relapse‐free survival curves of ESCC patients of cohort 3 (*n* = 100) stratified by low and high FBL expressions from IHC staining. Error bars represent mean ± SEM. ^*^ *p* < .05, ^**^ *p* < .01, ^***^ *p* < .001, two‐tailed Student's *t*‐test

Subsequently, we enrolled an ESCC patient cohort 2 (*n* = 41, Table S3) and performed Western blot assays to validate the expression of the above seven proteins with prognostic potential in ESCC tissues. The results showed that FBL, XPNPEP3, and BPTF were remarkably increased in ESCC tumors as relative to paired NATs (Figure 8A). By contrast, PCNP was not altered, while RBM3, MRPL14, and HACD2 were not detected in human ESCC tumors (Figure S15A). These results manifested the inconsistency between some mRNAs and their protein products, and further highlighted the active posttranscriptional modulation in ESCC. Furthermore, a previously reported gene expression dataset GSE23400,^28^ which contained 53 pairs of ESCC tumor tissues and matched NAT tissues of patients, was enrolled for re‐analysis. The result verified that *FBL* transcription was dramatically elevated in ESCC tumors (Figure S15B).

We then explored the impact of FBL, BPTF, and XPNPEP3 on the malignancy of ESCC cells. Two human ESCC cell lines KYSE150 and Eca109 were employed to delete *FBL*, *BPTF*, and *XPNPEP3* individually by using two distinct guide RNAs for each gene. In vitro studies revealed that *FBL* abrogation dramatically downregulated the expression of a cell proliferation marker PCNA and remarkably restrained ESCC cell growth (Figure 8B). Moreover, *FBL* ablation in ESCC cells elicited G1 phase arrest and restrained the expression of cyclin D1 key for G1/S transition, whereas its abrogation did not influence cell apoptosis (Figure S15C–E). In addition, *FBL* deletion in KYSE150 cells remarkably repressed the activity of PI3K/AKT signaling, as demonstrated by the downregulation of phosphorylated AKT at Thr308 and Ser473, respectively (Figure S15F). The role of PI3K/AKT signaling in promoting G1/S transition has been previously well established.^47^ Indeed, suppression of PI3K/AKT signaling by using a PI3K inhibitor LY294002 caused increased G1 phase arrest in KYSE150 cells (Figure S15G,H). In vivo studies revealed that *FBL* deletion markedly impaired the neoplastic growth of KYSE150 xenograft tumors (Figure 8C,D). However, ablation of *BPTF* or *XPNPEP3* did not influence ESCC cell propagation (Figure S15I,J). Together, these results demonstrated that *FBL*, but not *BPTF* and *XPNPEP3*, was essential for ESCC cell growth via activation of PI3K/AKT signaling and promotion of G1/S transition.

Finally, we enrolled an ESCC patient cohort 3 (*n* = 100, Table S4) to conduct an IHC staining assay study of FBL, and results confirmed its upregulated expression in ESCC tumors relative to NATs, as well as its usefulness as a prognostic biomarker (Figure 8E). Of importance, high FBL expression predicted inferior overall survival and relapse‐free survival of patients with ESCC (Figure 8F). Notably, analysis of the TCGA RNA‐seq data showed that high transcription of *FBL* was not associated with dismal overall survival of patients with ESCC (*n* = 78, Figure S15K), indicating no relevance between *FBL* mRNA and ESCC patient prognosis. Collectively, these findings demonstrated that high expression of FBL at protein level in tumor tissues was indicative of poor prognosis of ESCC patients.

### 3.7 Recapitulation of key molecular events participating in ESCC development

**FIGURE 9:**
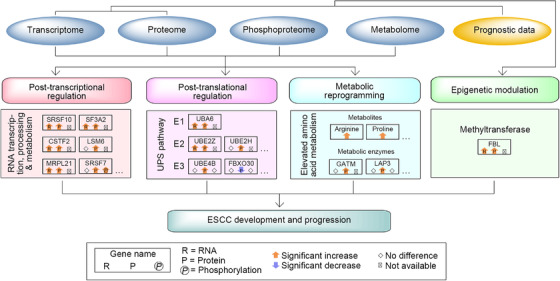
A model depicting molecular events during ESCC development identified by the multilayer study. Altered molecular events of epigenetic modulation, posttranscriptional and posttranslational regulation, and metabolic reprogramming, which were potentially involved in ESCC development and progression, are depicted

As mentioned above, FBL was crucial for ESCC cell growth in vitro and in vivo. FBL is a nucleolar methyltransferase that mainly functions in site‐specific methylation of rRNA and histone H2A, thus promoting ribosome assembly and early embryonic development.^48^, ^49^ Hence, its increased presence in tumors indicated that epigenetic modulation and mRNA translation in ribosomes may be involved in ESCC development. Second, as an important posttranscriptional process, AS was active in ESCC tumors (Figure 3F). Moreover, pathways related to RNA transcription, processing, and metabolism were stimulated in ESCC tumors (Figure 6). Additionally, UPS, a key pathway for proteolysis, was stimulated in ESCC tumors (Figures 3A,E and S9). Together, these results indicated that posttranscriptional and posttranslational regulation participated in ESCC development. Finally, a series of metabolites and metabolic pathways were upregulated in ESCC tumors (Figure 4D,E), implying that activated metabolism was required for sustaining the malignancy of ESCC. In summary, we inferred that molecular events in epigenetic, posttranscriptional, posttranslational, and metabolic layers cooperated closely to promote ESCC development and progression (Figure 9).

### 3.8 Tumor antigens and drug annotation of potential cancer drivers and key kinases

**FIGURE 10:**
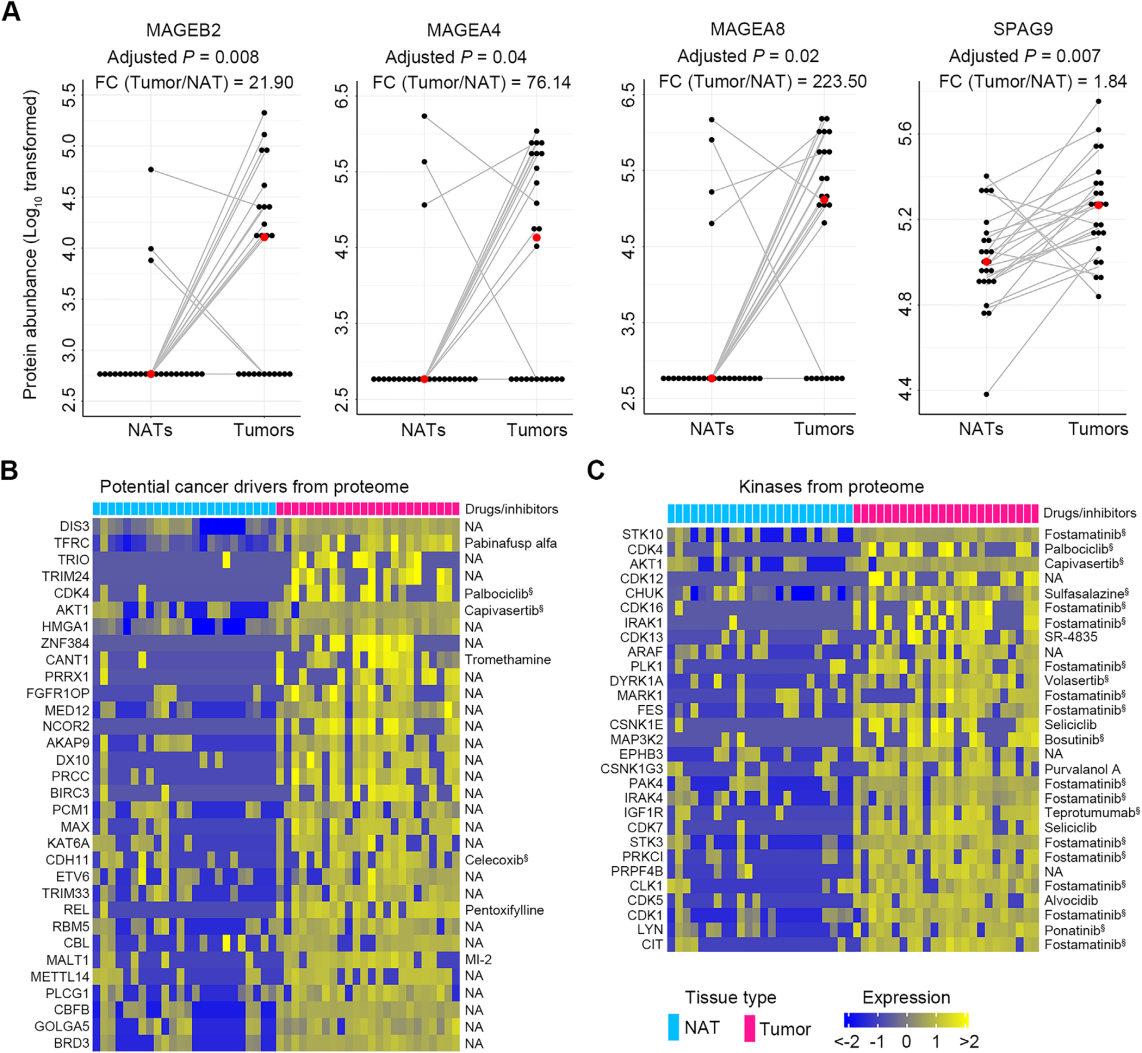
Identification of tumor antigens and drug annotation of potential cancer drivers and key kinases in ESCC. (A) Four tumor antigens with significant increases in ESCC tumors were identified by comparison of our proteomic data to a cancer/testis antigen list downloaded from the CTDatabase. Red dots represent the median values of each group. *p*‐Values were computed using the Wilcoxon rank‐sum test with Bonferroni correction. (B) A total of 32 potential cancer drivers with more than four‐fold increase in ESCC tissues were identified by comparison of our proteomic data to a list of potential cancer drivers provided by a previous study. (C) A total of 29 kinases with more than four‐fold increase in ESCC tissues were discovered by comparison of our proteomic data to a kinase list from databases, PhosphoSitePlus and NetworKIN. Identified cancer drivers and key kinases were annotated with targeted drugs or inhibitors by searching the databases, DrugBank and PKIDB. ^§^Drugs or inhibitors approved by Food and Drug Administration for clinical trial. NA, not available

Identification of new tumor antigens including CT antigens would afford more opportunities for vaccine development for cancer immunotherapy.^50^ By using the proteomic data, four known CT antigens, MAGE family member B2 (MAGEB2), MAGE family member A4 (MAGEA4), MAGE family member A8 (MAGEA8), and sperm‐associated antigen 9 (SPAG9), were found to be significantly increased in ESCC tumors with FC range from 1.84 to 223.50 as compared with NATs (adjusted *p *< .05, FDR *q* < .05) (Figure 10A).

In addition, identification of altered potential cancer drivers and kinases would improve our understanding of cancer biology and give rise to new therapeutic targets.^21^, ^51^, ^52^ We used our proteomic data to conduct the investigation. By comparison of our data to a list of potential cancer drivers described previously,^21^ we identified 32 potential drivers with more than four‐fold increase in ESCC tissues (adjusted *p* < .05, FDR *q* < .05) (Figure 10B). Furthermore, by data comparison to a kinase list from PhosphoSitePlus and NetworKIN,^22^ we found 29 known kinases with more than a four‐fold increase in ESCC tissues (adjusted *p* < .05, FDR *q* < .05) (Figure 10C). Subsequently, two drug databases, DrugBank^23^ and PKIDB,^24^ were used for drug annotation for these potential cancer drivers and kinases elevated in ESCC. The results revealed that 21.88% (7/32) of cancer drivers and 86.21% (25/29) of kinases possessed targeted inhibitors (Figure 10B,C). These inhibitors could be tested as new therapeutics for ESCC.

## 4 DISCUSSION

Previous omics studies of ESCC mainly focused on elucidating the genomic aberrations of this malignancy.^3^, ^4^, ^5^, ^6^, ^7^, ^8^, ^9^ Notably, by conducting a comprehensive genomic analysis of 158 ESCC cases, Song et al. identified several new mutated genes as novel oncogenes of ESCC and also found a series of new gene mutations that were potentially involved in the activity regulation of histones and several essential signaling pathways.^53^ However, molecular perturbations in ESCC at posttranscriptional and posttranslational levels were not distinguished in these studies.

The current study provides a comprehensive characterization of the molecular systems of ESCC at the transcriptomic, proteomic, phosphoproteomic, and metabolomic levels. We demonstrated that expression patterns of genes, gene isoforms, proteins, phosphosites, and metabolites were all overtly altered in ESCC tumors relative to NATs. The conservation of modified expression patterns of genes, gene isoforms, and metabolites was verified in carcinogen‐induced ESCC mice. Of importance, integrative analysis of transcriptomic and proteomic data revealed a remarkable discrepancy between mRNAs and the corresponding proteins in ESCC tumors, hence identifying high activities of ESCC tumors in posttranscriptional and posttranslational processes, including RNA transcription, RNA AS, RNA decay, protein translation, and proteolysis. It is reported that posttranscriptional processes, such as RNA AS, RNA stability, and RNA decay, play a vital role in tumorigenesis and tumor progression.^37^, ^54^ For example, increased mRNA stability of *SEMA4D* regulated by HuR promotes cell proliferation and migration of ESCC cells.^55^ In the current study, we found that a panel of 480 genes with high expression both at RNA and protein levels, together with a series of differential proteins discovered by proteomic profiling, were potentially involved in modulating posttranscriptional and posttranslational processes of ESCC. Notably, among these proteins, those with extremely high expression in ESCC tumors included SRSF10, SF3A2, CSTF2, RIF1, LSM6, MRPL21, and UBE2A. SRSF10, a well‐known splicing factor, mediates AS of interleukin 1 receptor accessory protein (IL1RAP) and promotes tumorigenesis in the cervix.^56^ SF3A2 and CSTF2 are RNA‐binding proteins.^21^ SF3A2 stimulates inclusion of exon 14 of the histone methyltransferase enhancer of zeste 2 polycomb repressive complex 2 subunit (EZH2) and has pro‐proliferative activity in renal cancer.^57^ CSTF2 induces 3′‐UTR shortening of Rac family small GTPase 1 (RAC1) to exacerbate cellular malignancy in urothelial carcinoma.^58^ RIF1 modulates replication timing regulation and activates expression of human telomerase reverse transcriptase to expedite epithelial ovarian cancer growth.^59^ LSM6 and MRPL21 are RNA‐binding proteins.^21^ LSM6 participates in reducing E‐cadherin expression, thus promoting cell migration in breast cancer.^60^ MRPL21 is highly expressed in several cancers and could be used as a biomarker for cancer prediction.^61^ UBE2A, an E2 ubiquitin‐conjugating enzyme, involved in DNA damage repair by catalyzing the ubiquitination of different target proteins, promotes cell cycle progression and tumorigenesis.^62^ Due to the well‐established tumorigenic roles of these proteins, it is reasonable to hypothesize that active posttranscriptional and posttranslational regulation is potential oncogenic driver of ESCC.

To the best of our knowledge, the present study is the first to integrate proteomic, phosphoproteomic, and metabolomic data to thoroughly portray the perturbations of signaling and metabolic pathways in ESCC. Analyses of proteomic and phosphoproteomic data revealed that pathways involved in RNA metabolism, transcription, and translation were enhanced at both protein and phosphorylation levels, thus underscoring posttranscriptional processes as possible etiological determinants of ESCC. There is increasing, consistent evidence to corroborate the importance of this biological process in ESCC development and progression.^63^, ^64^ Future investigations are necessary to complete our understanding of the biological mechanisms that determine ESCC malignancy. Additionally, our proteomic and metabolomic data revealed an enhanced amino acid metabolism in ESCC tumors, indicating an addiction of ESCC cells to amino acids. Indeed, previous studies have found that a series of amino acids were remarkably upregulated in ESCC tumors.^65^, ^66^ Intriguingly, we observed that several metabolites were positively linked to an ESCC stemness marker ALDH1, indicating a close relationship between metabolism and ESCC stemness. Further work is required to ascertain the biological functions and translational potential of these abnormal metabolic pathways and metabolites in ESCC.

An essential finding of this study is the identification of crucial proteins with prognostic potential for ESCC. For 66 of them, which are negatively linked to patient survival, their prognostic value is worthy of a validation in future independent cohorts. It should be particularly noted that this study yields a newly identified ESCC prognostic marker, FBL, a nucleolar methyltransferase that mainly functions in site‐specific methylation of rRNA and histone H2A, ribosome assembly, and early embryonic development.^48^, ^49^, ^67^, ^68^ FBL is involved in rDNA synthesis during the interphase of the cell cycle, and is required for normal nuclear morphology and cancer cell growth.^49^, ^68^ To the best of our knowledge, there are no published studies reporting the role of FBL in ESCC. Here, we found that FBL is highly expressed in ESCC tissues, negatively associated with patient prognosis, and vital for ESCC cell growth via stimulation of PI3K/AKT signaling and promotion of cell cycle progression, indicating that FBL is a potential therapeutic target against ESCC. The underlying molecular mechanism of how FBL regulates PI3K/AKT signaling requires further investigation.

Finally, therapeutic indications from this study should be evaluated. First, molecular pathways involved in posttranscriptional and posttranslational regulation are potential new therapeutic targets for ESCC treatment. For example, seven of these pathway proteins identified by GO and GSEA analyses, including SRSF10, SF3A2, CSTF2, RIF1, LSM6, MRPL21, and UBE2A, are extremely highly expressed in ESCC and have well‐established tumor‐promoting roles, thus supporting them as latent targets for curative treatment of ESCC. In addition, UPS, a key pathway involved in posttranslational regulation, is an appealing target for cancer therapy.^69^ Our proteomic data reveals that many UPS enzymes are dramatically upregulated in ESCC tumors, implying that inhibitors of UPS may be useful against ESCC. In line with this reasoning, a previous study reported that a well‐known proteasome inhibitor, bortezomib was able to induce cell cycle arrest and cell apoptosis, thus potentiating cytotoxicity of radiation therapy for ESCC.^70^ Second, those prognosis‐associated pathways enriched by KEGG analysis could be exploited as therapeutic targets for ESCC. For instance, one of the inhibited pathways, the p53 signaling pathway, drives the oncogenesis of ESCC as previously reported.^25^ Third, due to four CT antigens with an overt increase in ESCC tumors, it may be possible to develop vaccines to specifically eliminate ESCC cells. Finally, based on the drug annotation for potential cancer drivers and key kinases identified by our proteomic data, we can test the efficacy of these inhibitors in preclinical ESCC models as well as clinical trials in the future and develop new attractive therapeutics for the treatment of ESCC.

## 5 CONCLUSIONS

By using a multi‐omics approach, we deciphered new molecular events involved in ESCC development and progression, including aberrant methyltransferase expression, hyperactive posttranscriptional and posttranslational regulation, and reshaped metabolism. These findings have deepened our understanding of ESCC pathobiology and provided new prognostic biomarkers for risk stratification of ESCC patients. Furthermore, these findings unveiled new therapeutic targets and strategies for ESCC.

## CONFLICT OF INTEREST

The authors declare that there is no conflict of interest.

## ETHICS STATEMENT

All participants provided informed written consent in accordance with the regulation of the Institutional Review Board of the Affiliated Tumor Hospital of Nantong University (Approval number 2019‐022) in agreement with the Declaration of Helsinki. Studies of carcinogen‐induced ESCC mice were approved and conducted by Wuhan Servicebio Technology Co., Ltd (Approval number 2018015). Subcutaneous tumor xenograft experiments were approved by the Institutional Animal Care and Use Committee of Longhua Hospital (Approval number LHERAW‐19038).

## AUTHORS CONTRIBUTIONS

Conceptualization: Wen‐Lian Chen, Wei Wang, and Wei Jia. Multilayer omics study, data mining, and bioinformatics analysis: Wen‐Lian Chen, Hua Li, and Yan Ni. Pathology, H&E, and IHC staining assays: Xiaoxia Jin and Guanzhen Yu. Molecular biology and in vitro and in vivo studies: Xin Jing, Jia Wu, Dan Liu, Jing Yang, and Qin Luo. Patient enrollment, tissue samples, and clinical data: Lei Liu, Jia Wu, Fengying Wang, Minxin Shi, Haimin Liu, Jibin Liu, and Wei Wang. Manuscript writing: Wen‐Lian Chen. Writing ‐ review and editing: Lijun Jia, Hua Li, and Wei Jia.

## Supporting information

SUPPORTING INFORMATION

## Data Availability

Our raw data have been deposited to the National Omics Data Encyclopedia (NODE) database at Bio‐Med Big Data Center affiliated with Shanghai Institute of Nutrition and Health, Chinese Academy of Sciences, with project IDs of OEP002359 for RNA‐seq dataset, OEP002405 for proteomic dataset, OEP002366 for phosphoproteomic dataset, and OEP002347 for metabolomic dataset. The web link for NODE database is https://www.biosino.org/node.
