# Supplementary material for: A multi‐omics study delineates new molecular features and therapeutic targets for esophageal squamous cell carcinoma
Source: Clin Transl Med. 2021 Sep 26;11(9):e538. doi: 10.1002/ctm2.538 (PMC8473482; doi:10.1002/ctm2.538)
Supplement: Supplementary file 1 — SUPPORTING INFORMATION [file CTM2-11-e538-s001.doc]

**Supporting information**

**A multi-omics study delineates new molecular features and therapeutic targets for esophageal squamous cell carcinoma**

Xing Jin^1,7^, Lei Liu^2,7^, Jia Wu^1,7^, Xiaoxia Jin^3^, Guanzhen Yu^1^, Lijun Jia^1^, Fengying Wang^1^, Minxin Shi^2^, Haimin Lu^2^, Jibin Liu^2^, Dan Liu^1^, Jing Yang^1^, Hua Li^4^, Yan Ni^5^, Qin Luo^1^, Wei Jia^6^, Wei Wang^2^, Wen-Lian Chen^1^

^1^Cancer Institute, Longhua Hospital Shanghai University of Traditional Chinese Medicine, Shanghai 200032, China

^2^Department of Thoracic Surgery, the Affiliated Tumor Hospital of Nantong University, Nantong 226361, China

^3^Department of Pathology, the Affiliated Tumor Hospital of Nantong University, Nantong 226300, China

^4^Bio-ID Center, School of Biomedical Engineering, Shanghai Jiao Tong University, Shanghai 200240, China

^5^The Children’s Hospital, Zhejiang University School of Medicine, National Clinical Research Center for Child Health, Hangzhou 310029, China

^6^Hong Kong Traditional Chinese Medicine Phenome Research Center, School of Chinese Medicine, Hong Kong Baptist University, Kowloon Tong 999077, Hong Kong, China

^7^These authors contributed equally to this work.

**Correspondence**

Wei Jia, Hong Kong Traditional Chinese Medicine Phenome Research Center, School of Chinese Medicine, Hong Kong Baptist University, Kowloon Tong 999077, Hong Kong, China.

Email: weijia1@hkbu.edu.hk

Wei Wang, Department of Thoracic Surgery, the Affiliated Tumor Hospital of Nantong University, Nantong 226361, China.

Email: wangweimz@126.com

Wen-Lian Chen, Cancer Institute, Longhua Hospital Shanghai University of Traditional Chinese Medicine, Shanghai 200032, China.

Email: chenwl8412@shutcm.edu.cn

**Supporting materials and methods**

**Patient enrollment and clinical sample acquisition**

In this study, three independent patient cohorts were enrolled from the Affiliated Tumor Hospital of Nantong University (ATHNU) (Nantong, Jiangsu Province, China) at different time periods (from 2013 to 2018 for cohort 1, from 2018 to the present for cohort 2 and from January, 2012 to December, 2012 for cohort 3). Cohort 1, cohort 2 and cohort 3 contained 24, 41 and 100 newly-diagnosed patients respectively, and these patients received no prior treatment for their disease. All cases had ESCC histology but were collected regardless of histologic grade or surgical stage. Cases were staged according to the American Joint Committee on Cancer 8^th^ edition staging system. [^1^](#_ENREF_1)

In ATHNU, ESCC tumor and matched normal adjacent tissue (NAT) samples were collected from patients when they underwent esophagectomy. Tissues were either flash frozen in liquid nitrogen or formalin-ﬁxed parafﬁn-embedded. Hematoxylin and eosin (H&E) stained slides were examined for quality assessment. Routinely, several tissue segments from each case were harvested. Each case was independently reviewed by three board-certificated pathologists to determine the pathology. Acceptable ESCC tumor tissue segments were determined by the pathologists based on the percent viable tumor nuclei (> 80%), total cellularity (> 50%) and necrosis (< 20%). Specimens from cohort 1 and cohort 2 were flash frozen in liquid nitrogen and then transferred to a -80 ℃ freezer until approval for shipment to the Cancer Institute, Longhua Hospital (CILH, Shanghai, China). Specimens from cohort 3 were formalin-ﬁxed parafﬁn-embedded and used for tissue microarray preparation and immunohistochemistry (IHC) assay as we previously described. [^2^](#_ENREF_2)

Specimens from cohort 1 and cohort 2 were shipped using dry ice that maintained a temperature around -80℃ to CILH. Receipt of specimens at CILH included a physical inspection and review of the remaining dry ice for specimen integrity, followed by entry into a biospecimen tracking database. Specimens were again stored in -80 ℃ freezer until further processing.

Tissues from cohort 1 and cohort 2 were cryopulverized and divided into aliquots for RNA, protein and metabolite extraction. Specimens from cohort 1 were used for multi-layer omics measurements, while specimens from cohort 2 and cohort 3 were used for further validation. Tissue aliquots from cohort 1 were shipped to RNA-seq (Cloudseq Biotech Inc., Shanghai, China), proteomic (Applied Protein Technology lnc., Shanghai, China) and metabolomic (Metabo-Proﬁle Inc., Shanghai, China) characterization centers in dry ice shippers. A shipment manifest accompanied all distributions for the receipt and integrity inspection of the specimens at the destination.

Overall survival (OS) and relapse-free survival (RFS) were acquired for patient cohort 1 and cohort 3. OS was defined as time from the date of surgery to the date of death from any cause or the last follow-up. RFS was defined as time from the date of surgery to the date of first disease recurrence or the last follow-up.

**Carcinogen-induced ESCC mouse model and esophageal sample collection**

A known carcinogen 4-nitroquinoline 1-oxide (4-NQO, Sigma, cat#N8141) was used to induce ESCC in six-week-old C57BL/6 female mice as previously reported.[^3^](#_ENREF_3) The study was approved and conducted by Wuhan Servicebio Technology Co., Ltd (Approval number 2018015). Briefly, 4-NQO stock solution was prepared in propylene glycol at 6 mg/mL and then divided into aliquots for -20 ℃ storage. The experiment was carried out using two groups of mice. The experimental group was administered the carcinogen via drinking water containing 4-NQO with a final concentration of 100 μg/mL. While the control group was supplied with drinking water with no 4-NQO. Two groups received the same volume of propylene glycol in the drinking water, and drinking water was changed once a week. After a 16-week carcinogen or vehicle treatment, mice were followed-up for another 12 weeks.

At the end of the experiment, all mice were killed and their esophageal tissues were collected. Specimens were either flash frozen in liquid nitrogen or formalin-ﬁxed parafﬁn-embedded. H&E stained slides were examined for cancerous lesion assessment. Additionally, parafﬁn-embedded tissues were prepared for IHC assay for expression analysis of keratin 14 (Abcam, cat#ab7800) and Ki-67 (Abcam, cat#ab245113). Flash-frozen specimens were transferred to a -80 ℃ freezer until approval for shipment to CILH. Cryostored specimens were shipped using dry ice that maintained a temperature around -80 ℃ to CILH. Receipt of specimens at CILH included a physical inspection and review of the remaining dry ice for specimen integrity, followed by entry into a biospecimen tracking database. Then specimens were cryopulverized and divided into aliquots for RNA and metabolite extraction. Tissue aliquots were shipped to RNA-seq and metabolomic characterization centers in dry ice shippers. Notably, RNA-seq (GENEFUND Inc., Shanghai, China) and metabolomic (Metabo-Proﬁle Inc., Shanghai, China) surveys were performed using two different batches of mice respectively because of the limited esophagus tissue of each mouse. A shipment manifest accompanied all distributions for the receipt and integrity inspection of the specimens at the destination.

**RNA-sequencing, extraction of alternative splicing data and data analysis**

***RNA-sequencing*** ***and data analysis***

RNA-sequencing (RNA-seq) was performed by Cloud-Seq Biotech Inc. (Shanghai, China) following our previous procedure.[^2^](#_ENREF_2) Total RNA was extracted from fresh frozen tissues using the Trizol reagent (Thermo Fisher Scientific, cat#15596018) and purified by removing rRNAs with NEBNext® rRNA Depletion Kit (New England Biolabs Inc., Massachusetts, USA, cat#E6310X) following the manufacturer's instructions. All RNA analytes were assessed for fragment size, integrity, purity and concentration.

Subsequently, RNA libraries were constructed by using NEBNext® Ultra^TM^ Ⅱ Directional RNA Library Prep Kit (New England Biolabs, Inc., Massachusetts, USA, cat#E6310) according to the manufacturer’s instructions. Libraries were controlled for quality and quantified using the Bioanalyzer 2100 system (Agilent Technologies, Inc., USA). Library sequencing was performed on an illumina Hiseq 4000 sequencer with 150 bp paired end mode. Raw reads were quality controlled by Q30. After removing sequencing adaptors, short reads (length < 35 bp) and low-quality reads with software of Cutadapt (v1.18) and Trimmomatic (v0.35), high-quality clean reads were obtained. Then, by using HISAT2 software (v2.1.0), clean reads of human data were aligned to the human genome (assembly GRCh38), while clean reads of mouse data were aligned to the mouse genome (assembly GRCh38). For the raw count data of genes and gene isoforms, R package edgeR (v3.26.8)[^4^](#_ENREF_4) was used to filter lowly expressed items and to execute normalization to get reads per kilobase per million (RPKM) expression values. The raw RNA-seq data were deposited to The National Omics Data Encyclopedia (NODE) database at Bio-Med Big Data Center (BMBDC) affiliated with Shanghai Institute of Nutrition and Health (SINH), Chinese Academy of Sciences (CAS) with a Project ID of OEP002359. Differentially expressed mRNAs were identified by the cuts of *P* < 0.05, FDR q < 0.05 and fold change (FC) > 1.5 or FC < -1.5.

***Extraction and analysis of alternative splicing data***

Seven common alternative splicing (AS) events were analyzed using SpliceSeq tool[^5^](#_ENREF_5) in this study, including alternate acceptor site (AA), alternate donor site (AD), alternate promoter (AP), alternate terminator (AT), exon skip (ES), mutually exclusive exons (ME) and retained intron (RI). RNA-seq data in FASTQ format were loaded into SpliceSeq tool to implement analysis. To evaluate how efﬁciently sequences were spliced into transcripts for a specific event, percent-splice-in (PSI) values, which were the ratio of normalized read counts indicating inclusion of a transcript element over the total normalized reads for that event (both inclusion and exclusion reads), were calculated for each RNA transcript of each sample as reported previously. Statistically different AS events between NAT and tumor tissues were defined as *P* < 0.05, |mean PSI of NAT samples – mean PSI of tumor samples| > 0.1, mean RPKM of NAT samples ≥ 1, mean RPKM of tumor samples ≥ 1, and magnitude ≥ 1.

**MS sample processing for proteome and phosphoproteome**

***Protein extraction and digestion***

Total proteins were extracted by tissue homogenization on a MP FastPrep-24 homogenizer (24×2,6.0M/S, 60 sec, twice) in SDT buffer (4% SDS, 10 mM 1,4-dithiothreitol (DTT), 150 mM Tris-HCl, pH 8.0). The lysates were further sonicated and denatured at 95 ℃ for 15 min. The insoluble debris was removed be centrifugation at 14,000 × g for 40 min, and the supernatant was quantified by using a BCA Protein Assay Kit (Thermo Fisher Scientific, cat#23227) and then run by SDS-PAGE electrophoresis for quality assessment. An equal aliquot from each sample in this experiment was pooled into one sample for data dependent acquisition (DDA) library generation and quality control (QC).

All protein samples along with pooled samples were digested using the ﬁlter-aided sample preparation (FASP) procedure.[^8^](#_ENREF_8) Briefly, 200 μg proteins were loaded in 10kDa centrifugal filter tubes (Millipore, cat#MRCPRT010), washed twice with 200 μL UA buffer (8 M urea in 150 mM Tris-HCI, pH 8.0), alkylated with 100 μL 50 M iodoacetamide in UA buffer for 30 min in the darkness, washed thrice with 100 μL UA buffer again and finally washed twice with 100 μL 50 mM NH_4_HCO_3_. All of the above steps were each centrifuged at 14,000 × g at room temperature for 30 min. Finally, each protein suspension was digested with 2 μg trypsin (Promega, cat#V5111) in 40 μL 50 mM NH_4_HCO_3_ buffer for 16 hr at 37 ℃. After digestion, peptides were eluted using 40 μL 50 mM NH_4_HCO_3_ buffer by centrifugation. Each peptide content was estimated by UV light spectral density at 280 nm. Digested peptides from 24 pairs of ESCC tumor and NAT samples were used for data-independent acquisition (DIA)-based LC-MS/MS analysis.

***Peptide fractionation for data-dependent acquisition library generation of proteome***

The DIA approach was used to perform proteomic measurements in this study; hence a DDA library was established first. Each digested, pooled sample (100 μg) was fractionated into 10 fractions using a Pierce™ High pH Reversed-Phase Peptide Fractionation Kit (Thermo Fisher Scientific, cat#84868). Each fraction was concentrated by vacuum centrifugation and reconstituted in 10 µL of 0.1% (vol/vol) formic acid. Concentrations of collected peptides from each fraction were determined by UV light spectral density at 280 nm. The iRT-Kits (Biognosys, cat#Ki-3002-2) was added to correct the relative retention time differences between runs with a volume proportion of 1:3 for iRT standard peptides *versus* collected peptides. Subsequently, each mixture of iRT standard peptides and collected peptides was prepared in the same way for the LC-MS/MS assay for DDA library generation.

***iTRAQ labelling and phosphopeptide enrichment for phosphoproteomic study***

For the phosphoproteomic assay, digested peptides from three pairs of ESCC tumor and NAT samples were desalted on C18 Cartridges (Empore™ SPE Cartridges C18 (standard density), bed I.D. 7 mm, volume 3 mL) (Merk, cat#66872-U) and reconstituted in 40 µL of 0.1% (vol/vol) formic acid. Concentrations of the reconstituted peptide solutions were measured by UV light spectral density at 280 nm. Then, 100 μg of desalted peptides from each sample were labelled with multiplex iTRAQ reagents (AB Sciex, Foster City, CA, cat#4352135) according to the manufacturer’s instructions. Labelled peptides were used for phosphopeptide enrichment with a High-Select™ Fe-NTA Phosphopeptide Enrichment Kit (Thermo Fisher Scientific, cat#A32992) according to the manufacturer’s instructions. Enriched peptides were vacuum-dried, reconstituted in 20 µL of 0.1% (vol/vol) formic acid and injected into a LC-MS/MS for analysis.

**Multivariate statistical analysis**

***Unsupervised hierarchical clustering and principal component analysis***

Unsupervised hierarchical clustering was performed using an R package ComplexHeatmap.[^9^](#_ENREF_9) The distance between each pair of samples was computed using Manhattan method, while hierarchical clustering of samples was implemented using the ward.D method. Principal component analysis (PCA) was performed using the function of prcomp in R (version 3.6.1), and PCA visualization was executed with a function of ggscatterhist provided by an R package of ggpubr.

***Integrative clustering***

To ascertain whether tissues with the same histological feature (ESCC or NAT) shared molecular signatures across platforms, iCluster,[^10^](#_ENREF_10) an integrative clustering approach was used. The R package iClusterPlus (version 1.22.0) was used to carry out the calculation.

***Multi-block discriminant analysis***

To further explore whether the differences between ESCC and NAT samples were consistent at distinct molecular layers, we used the approach of Data Integration Analysis for Biomarker discovery using a Latent cOmponent (DIABLO)[^11^](#_ENREF_11) to conduct multi-block discriminant analysis. The function of block.splsda in R package mixOmics[^11^](#_ENREF_11) was used for computation.

**Multi-omics analysis**

***mRNA-protein correlation***

A total of 6,174 genes with complete mRNA and protein data across all 48 ESCC and NAT samples were used to calculate gene-wise and sample-wise mRNA and protein correlations. Spearman correlation was conducted for each mRNA-protein pair across tumors and NATs and for each individual sample across 6,174 genes. Density ridgeline plots were generated using R packages ggplot2 and ggridges.[^12^](#_ENREF_12)

***Integrative network analysis of proteomic and phosphoproteomic data***

By analyzing the proteomic and phosphoproteomic data, differentially expressed proteins and phosphosites in ESCC tumors involved in pathways of RNA transcription, processing and metabolism were visualized with Cytoscape.[^13^](#_ENREF_13)

***Integrative network analysis of proteomic and metabolic data***

Integrative analysis of metabolic enzymes from proteomic data and metabolites from metabolomic data was executed using a Cytoscape App MetScape 3.[^14^](#_ENREF_14) The most perturbed metabolic pathway in ESCC, arginine and proline metabolism, was selected for presentation. In addition, Spearman correlation between differentially expressed metabolites and phenotypic protein markers was visualized by heat map. Metabolites with significant correlation to at least one phenotypic protein marker were selected for exhibition.

**Download and analysis of the public TCGA RNA-seq and GEO microarray datasets**

TCGA RNA-seq dataset and GEO gene expression dataset GSE23400 for ESCC patients were downloaded from The Cancer Genome Atlas (https://cancergenome.nih.gov/) and the NCBI Gene Expression Omnibus (http://www.ncbi.nlm.nih.gov/geo/) respectively.

There were 78 ESCC patients in the TCGA dataset, whereas only eight patients possessed matched tumorous and NAT tissues. The remaining cases only possessed tumorous tissues. RNA-seq data of this dataset were normalized using FPKM method by which an expression value was normalized by taking into account each protein-coding gene length and the number of reads mappable to all protein-coding genes. To better explore the potential relevance between *FBL* mRNA levels and patient prognosis, 78 ESCC patients of this dataset were divided into two subgroups by using the upper quartile score of *FBL* as the cut-off value. Kaplan-Meier survival curves of these two subgroups were established to evaluate the prognostic potential of *FBL* mRNA levels.

GSE23400 dataset, which contained 53 pairs of ESCC and NAT tissues of patients, was normalized using the Robust Multichip Average method and then log_2_-transformed. Gene transcription of *FBL* between ESCC and NAT samples was compared using the non-parametric and paired two-class Wilcoxon rank-sum test. Notably, patient survival data were not available for this dataset.

**Functional experiments**

***Gene knockout using CRISPR-Cas9 approach***

Two human ESCC cell lines, KYSE150 (Stem Cell Bank, Chinese Academy of Sciences) and Eca109 (Stem Cell Bank, Chinese Academy of Sciences), were enrolled for gene knockout studies according to our previous description. Briefly, each guide RNA (gRNA) duplex was inserted into the lenti-Guide-CRISPR-v2-puro vector which was linearized by BsmBI endonuclease. The lentivirus was generated as follows: lenti-Guide-CRISPR-v2-puro vector containing nontarget control (NC) gRNA or lenti-Guide-CRISPR-v2-puro vector harboring target gRNA was co-transfected with psPAX2 and pMD2.G plasmids into HEK293T cell using the Lipofectamine 3000 reagent (Thermo Fisher Scientific, cat#L3000008) following the manufacturer’s instruction. Lentivirus-containing supernatants were harvested and filtered with 0.45 μm filter to remove cells. Subsequently, ESCC cells were infected with the lentivirus harboring NC gRNA or lentivirus harboring target gRNA in the presence of 8 µg/mL polybrene. Transfected cells were selected with puromycin for 48 hours. The following gRNA were used in this study:

gRNA of NC: 5’-AAGAAGAATTGGGGATGATG-3’;

gRNA#1 for *BPTF*: 5’-ACGCTATCTTTCAGATCAGC-3’;

gRNA#2 for *BPTF*: 5’-AGTCAATTAATTCTGCAAGT-3’;

gRNA#1 for *FBL*: 5’-CCTGTACACTCCCACGACCA-3’;

gRNA#2 for *FBL*: 5’-ATGTGATGGTGGAGCCGCAT-3’;

gRNA#1 for *XPNPEP3*: 5’-ACCATCCCAAAGTTCTCGAC-3’;

gRNA#2 for *XPNPEP3*: 5’-GGTCCGCGATCTGGCACTGA-3’.

***Cell viability assay***

Cells were cultured in in Dulbecco’s modified Eagle’s medium (Thermo Scientific, cat#12430054) supplemented with 10% fetal bovine serum (Thermo Scientific, cat#16140071). Cell viability was analyzed using a Cell Counting Kit-8 (Dojindo Laboratories, cat#CK04) according to the manufacturer’s recommendation.

***Subcutaneous tumor xenograft study***

The subcutaneous tumor xenograft study was conducted according to our previous protocol. Nontarget control cells and cells with *FBL* knockout were subcutaneously injected into the left and right hind flanks of 8-week-old female BALB/c-nude mice (Shanghai SLAC Laboratory Animal Co., Ltd, Shanghai, China). The tumor length and width were measured every 3 days using a caliper. Tumor sizes were computed as 0.5 × length × width^2^. At the end of the experiments, tumor xenografts were resected for imaging and weighing. Animal protocols and experiments were approved by the Institutional Animal Care and Use Committee of Longhua Hospital, Shanghai University of Traditional Chinese Medicine (Approval number LHERAW-19038).

**Western blot and immunohistochemistry staining assays**

Western blot was performed as previously described. Cells cultured *in vitro* were digested with 0.25% trypsin and lysed using RIPA buffer (Sigma-Aldrich, cat#R0278) containing 1% protease inhibitor cocktail (vol/vol, Sigma-Aldrich, cat#P8340) on ice. For tumor tissues, they were cut into small pieces and homogenized in RIPA buffer containing 1% protease inhibitor cocktail on ice. Supernatants of cell/tissue lysates containing total proteins were acquired by centrifugation at 12,000 ×g for 10 min and their concentrations were determined by using a BCA assay kit (Thermo Scientific, cat#23225). Protein extracts were boiled for 10 min together with sample loading buffer (Bio-rad, cat#1610747), resolved by SDS-PAGE, and then transferred to PVDF membranes. After incubation with primary antibodies overnight at 4 °C, the membranes were washed and then incubated with secondary antibodies conjugated with IgG-HRP (Cell Signaling Technology, cat#7074, cat#7076, cat#7077). The following primary antibodies was used in this study: HACD2 (PTPLB) (abcam, ab237026), RBM3 (proteintech, cat#14363-1-AP), MRPL14 (proteintech, cat#15040-1-AP), PCNP (proteintech, cat#11180-2-AP), XPNPEP3 (proteintech, cat#15655-1-AP), BPTF (abcam, ab72036), FBL (proteintech, cat#16021-1-AP), Cyclin D1 (Cell Signaling Technology, cat#2978), AKT (pan) (Cell Signaling Technology, cat#4691), phospho-AKT (S473) (Cell Signaling Technology, cat#3787), phospho-AKT (T308) (Cell Signaling Technology, cat#4056), and actin (Cell Signaling Technology, cat#4967S).

Immunohistochemistry staining was performed according to our previous protocol.[^2^](#_ENREF_2) Microtissue array from patient cohort 3 was stained using antibody against FBL or nonspecific IgG as negative control. The tissue sections were quantitatively scored based on the percentage of positive cells and staining intensity as described previously.[^17^](#_ENREF_17) Average percentage of positive cells were calculated in five areas of a given sample at a magnification of × 400 and scored from 0 – 100%. Staining intensity was scored as 0 for negative, 1 for weak, 2 for moderate, and 3 for strong. The proportion and intensity scores were then combined to acquire a weighted staining score for each case, ranging from 0 (0% of cells stained) to 3 (100% of cells stained). The upper quartile score value of FBL of ESCC tumors (n = 100) were used as the cut-off value to determine tumor tissues with low or high FBL staining.

**Cell cycle and cell apoptosis assays**

For cell cycle assay, ESCC cells were seeded in 6-well plates with 2 × 10^5^ cells per well and 2 mL complete medium per well. To analyze the impact of *FBL* deletion on cell cycle, gene modified cells were cultured for 48 hours. To assess the influence of PI3K/AKT activity on cell cycle, cells were treated by a PI3K inhibitor LY294002 for 48 hours. Next, cells were digested with 0.5% trypsin and washed by PBS for 2 times. The 70% cold ethanol was dropwise added to each cell suspension. Subsequently, cell suspensions were kept at -20 ℃ for 24 hours. Finally, cell cycle assay was performed using a cell cycle kit according to the manufacturer’s instruction.

For cell apoptosis analysis, ESCC cells were seeded in 6-well plates with 2 × 10^5^ cells per well and 2 mL complete medium per well. After culture of 48 hours, spent medium of each well was collected individually to harvest cells by centrifuging at 1500 rpm for 5 minutes, while adherent cells of each well were digested with 0.5% trypsin and harvested. For each well, cells from spent medium and adherent cells were combined together and then washed by PBS for 2 times. Finally, cell apoptosis assay was implemented on a flow cytometer using a cell apoptosis kit according to the manufacturer’s recommendation.

**Supplemental references:**

1. Amin MB, Greene FL, Edge SB, et al. The Eighth Edition AJCC Cancer Staging Manual: Continuing to build a bridge from a population-based to a more "personalized" approach to cancer staging. *CA Cancer J Clin*. 2017;67(2):93-99.

2. Chen WL, Jin X, Wang M, et al. GLUT5-mediated fructose utilization drives lung cancer growth by stimulating fatty acid synthesis and AMPK/mTORC1 signaling. *JCI Insight*. 2020;5(3).

3. Tang XH, Knudsen B, Bemis D, Tickoo S, Gudas LJ. Oral cavity and esophageal carcinogenesis modeled in carcinogen-treated mice. *Clin Cancer Res*. 2004;10(1 Pt 1):301-313.

4. Robinson MD, McCarthy DJ, Smyth GK. edgeR: a Bioconductor package for differential expression analysis of digital gene expression data. *Bioinformatics*. 2010;26(1):139-140.

5. Ryan MC, Cleland J, Kim R, Wong WC, Weinstein JN. SpliceSeq: a resource for analysis and visualization of RNA-Seq data on alternative splicing and its functional impacts. *Bioinformatics*. 2012;28(18):2385-2387.

6. Schafer S, Miao K, Benson CC, et al. Alternative Splicing Signatures in RNA-seq Data: Percent Spliced in (PSI). *Curr Protoc Hum Genet*. 2015;87:11 16 11-11 16 14.

7. Liu J, Li H, Shen S, et al. Alternative splicing events implicated in carcinogenesis and prognosis of colorectal cancer. *J Cancer*. 2018;9(10):1754-1764.

8. Wisniewski JR, Zougman A, Nagaraj N, Mann M. Universal sample preparation method for proteome analysis. *Nat Methods*. 2009;6(5):359-362.

9. Gu Z, Eils R, Schlesner M. Complex heatmaps reveal patterns and correlations in multidimensional genomic data. *Bioinformatics*. 2016;32(18):2847-2849.

10. Shen R, Olshen AB, Ladanyi M. Integrative clustering of multiple genomic data types using a joint latent variable model with application to breast and lung cancer subtype analysis. *Bioinformatics*. 2009;25(22):2906-2912.

11. Rohart F, Gautier B, Singh A, Le Cao KA. mixOmics: An R package for 'omics feature selection and multiple data integration. *PLoS Comput Biol*. 2017;13(11):e1005752.

12. Wickham H. ggplot2: Elegant Graphics for Data Analysis.: Springer-Verlag New York; 2016.

13. Shannon P, Markiel A, Ozier O, et al. Cytoscape: a software environment for integrated models of biomolecular interaction networks. *Genome Res*. 2003;13(11):2498-2504.

14. Karnovsky A, Weymouth T, Hull T, et al. Metscape 2 bioinformatics tool for the analysis and visualization of metabolomics and gene expression data. *Bioinformatics*. 2012;28(3):373-380.

15. Jin X, Liang Y, Liu D, et al. An essential role for GLUT5-mediated fructose utilization in exacerbating the malignancy of clear cell renal cell carcinoma. *Cell Biol Toxicol*. 2019;35(5):471-483.

16. Chen WL, Wang YY, Zhao A, et al. Enhanced Fructose Utilization Mediated by SLC2A5 Is a Unique Metabolic Feature of Acute Myeloid Leukemia with Therapeutic Potential. *Cancer Cell*. 2016;30(5):779-791.

17. Yu G, Yu W, Jin G, et al. PKM2 regulates neural invasion of and predicts poor prognosis for human hilar cholangiocarcinoma. *Mol Cancer*. 2015;14:193.


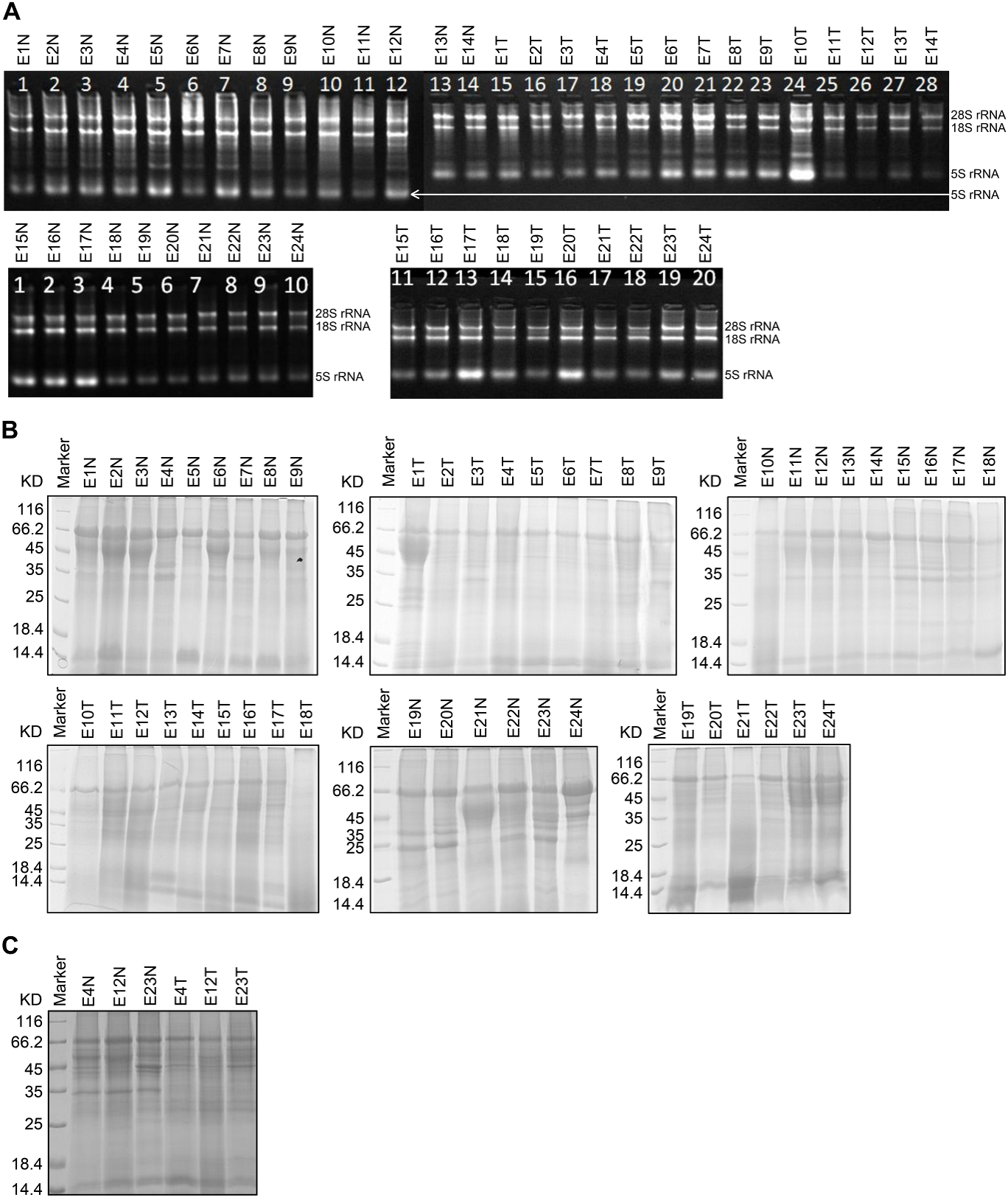


**Figure S1. Quality assessment of RNA and protein specimens from ESCC patient cohort 1.**

(**A**) Agarose gel electrophoresis assay for all 48 RNA samples from patient cohort 1. Notably, rRNA bands of most of these samples possessed 28S:18S rRNA ratios of 2:1, indicating good-quality of these RNA samples. (**B**) Sodium dodecyl sulfate-polyacrylamide gel electrophoresis was run for quality evaluation of all protein samples for proteomic investigation. The sharp bands for each sample indicated high quality of these protein specimens. (**C**) Sodium dodecyl sulfate-polyacrylamide gel electrophoresis was run for quality assessment of all protein samples of phosphoproteomic survey. The sharp bands for each sample indicated high quality for these protein specimens.


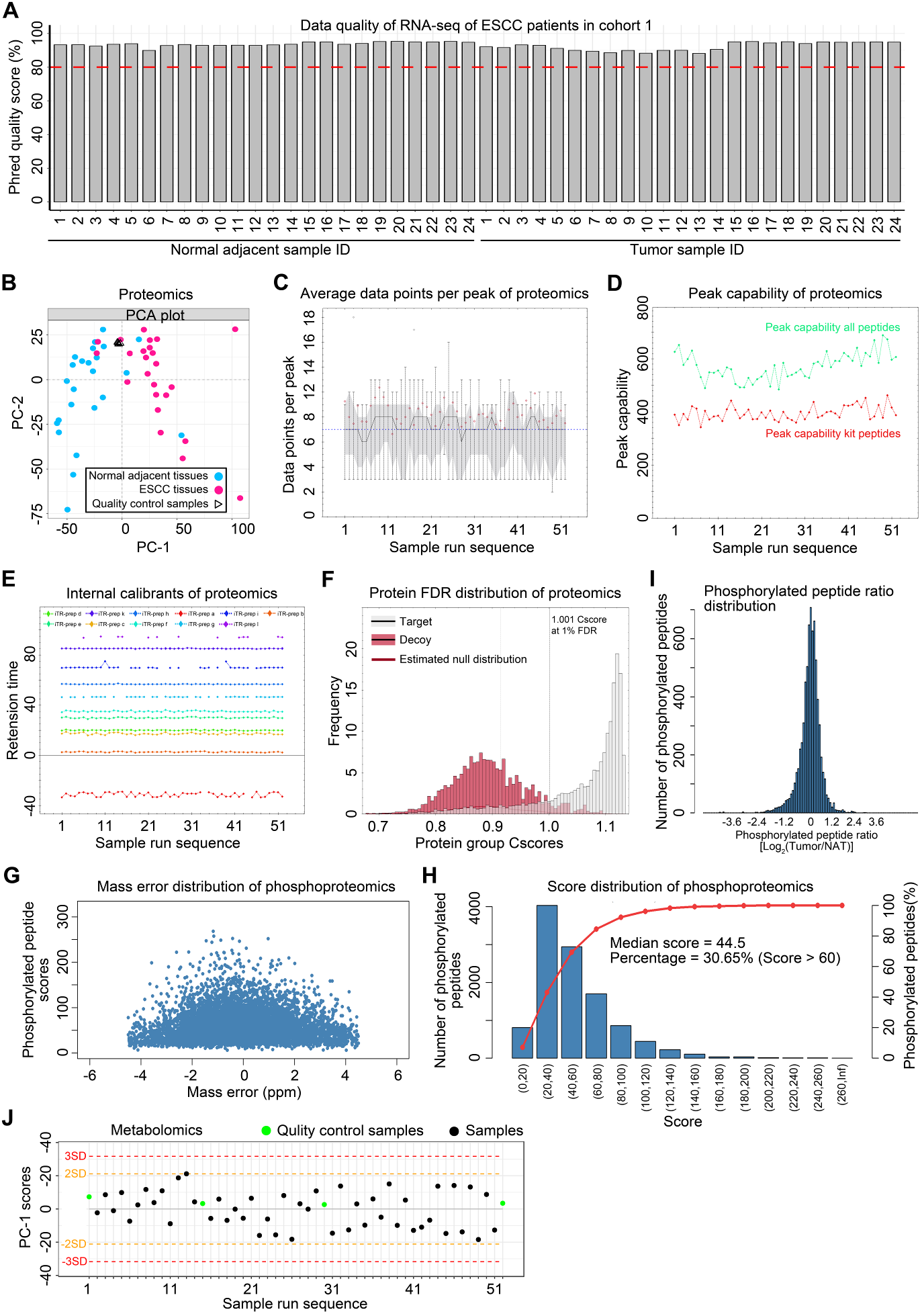


**Figure S2. Quality evaluation of omics data of ESCC patient cohort 1.**

(**A**) Phred quality scores of RNA-seq data across 48 samples from ESCC patient cohort 1. Notably, Phred quality scores of all samples were > 80%, indicating high quality of RNA-seq data. (**B**) Principal component analysis (PCA) of all protein samples along with quality control (QC) samples using proteomic data. All QC samples were clustered together, indicating very low systemic variation during measurement. (**C**) Data points for each peak of proteome. (**D**) Peak capability of all peptides and kit peptides of proteome. (**E**) Internal calibrants for the proteomic data. (**F**) Protein FDR distribution of proteomic data. (**G**) Mass error distribution of phosphoproteomic data. (**H**) Phosphorylated peptide score distribution of phosphoproteomic data. (**I**) Phosphorylated peptide ratio distribution of phosphoproteomic data. (**J**) Principal component analysis of all tested samples and QC samples using metabolomic data. Principal component 1 (PC-1) was selected for constructing the score plot. Low variation of PC-1 scores of all QC samples indicated high systemic stability during metabolomic measurement.


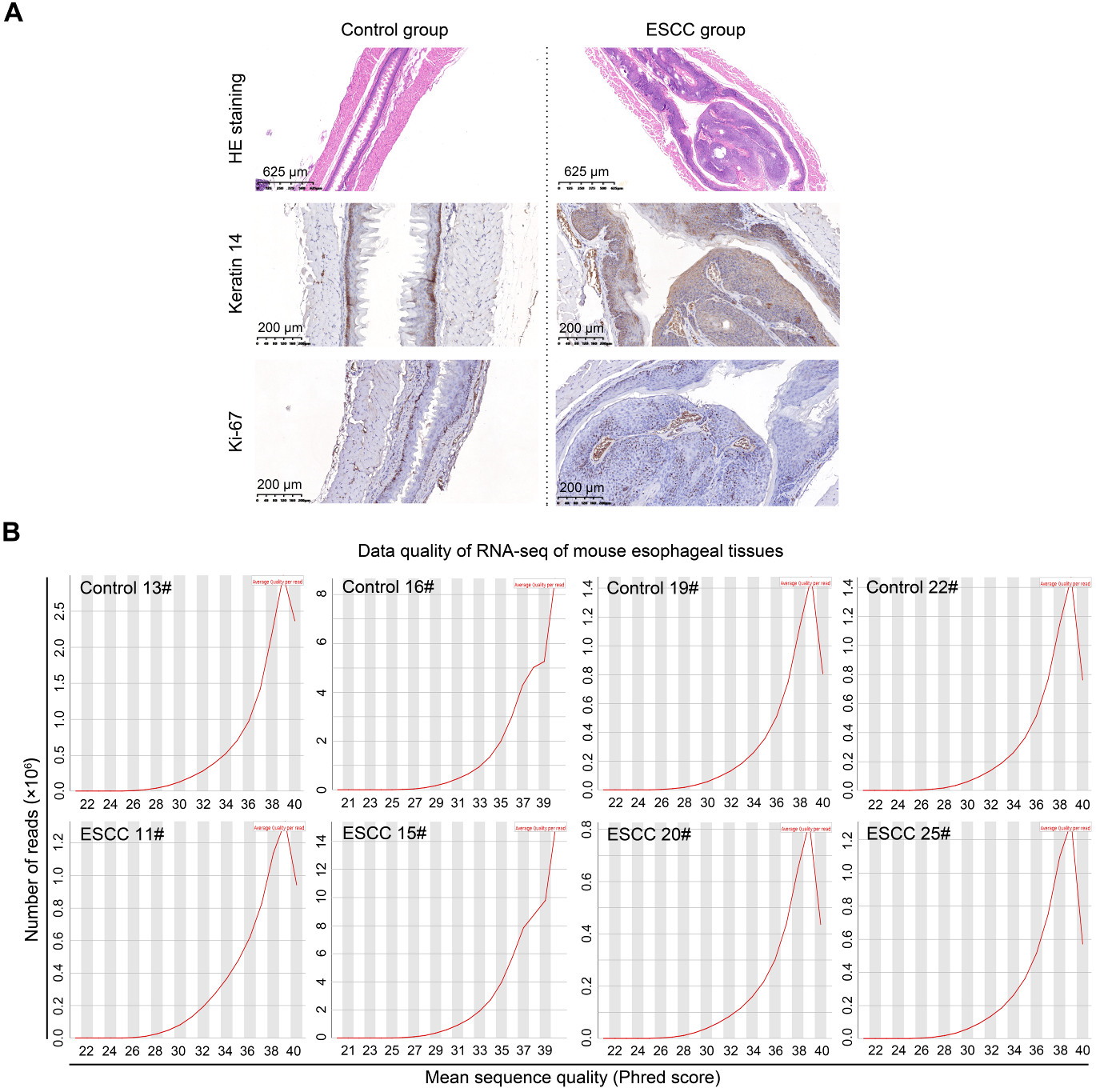


**Figure S3. Histological analysis of esophageal tissues and RNA-seq data quality of mice.**

(**A**) Representative histological images of esophageal tissues from ESCC and control mice respectively. H&E staining and IHC staining against keratin 14 and Ki-67 were exhibited. (**B**) Phred scores across all samples were analyzed to evaluate the RNA-seq data quality.


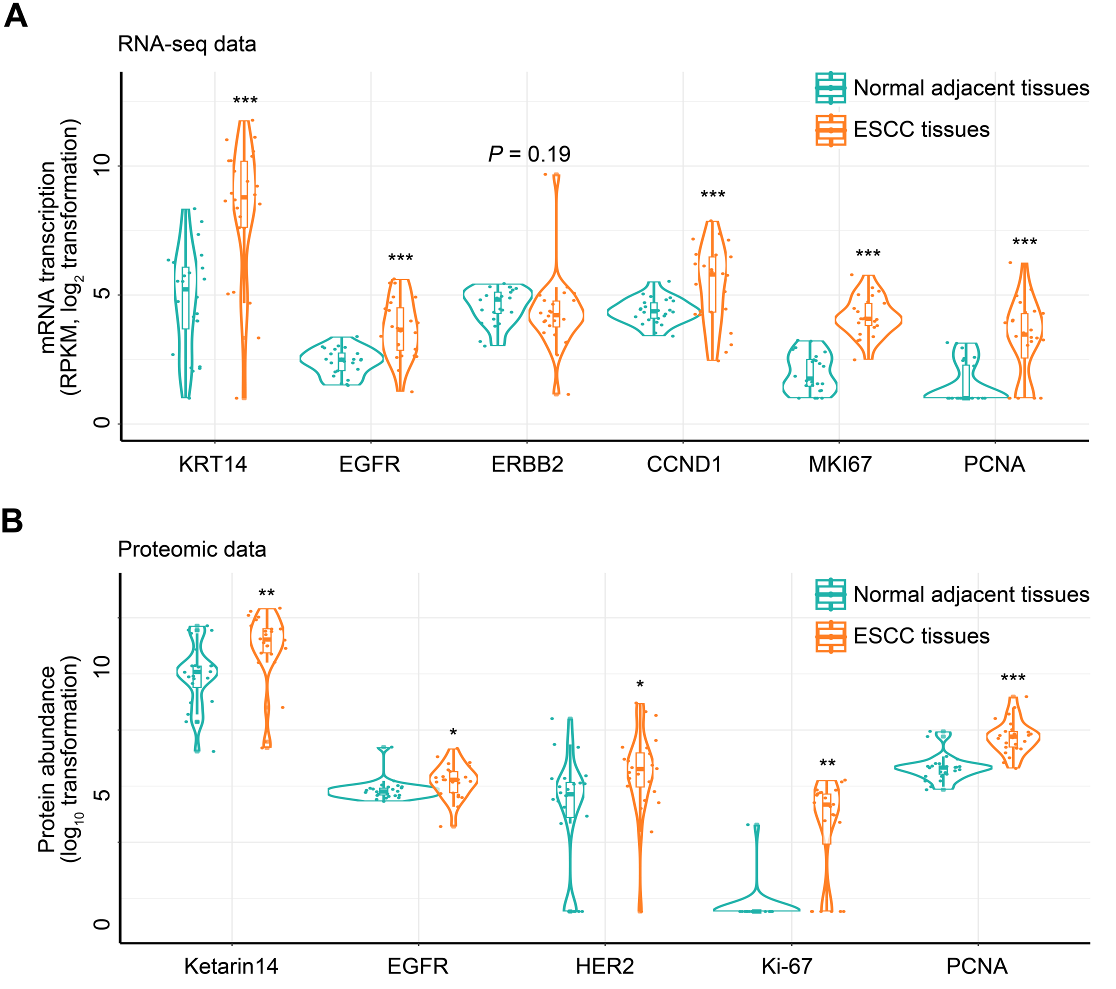


**Figure S4. Expression analysis of well-established ESCC markers in tissue samples of patient cohort 1.**

(**A**) Violin-box-scatter plots showing the transcription of genes *KRT14*, *EGFR*, *ERBB2*, *CCND1*, *MKI67* and *PCNA* between ESCC and NAT samples. Transcription data of these genes were derived from RNA-seq data. (**B**) Violin-box-scatter plots showing the abundance of proteins keratin 14, EGFR, HER2, Ki-67 and PCNA between ESCC and NAT samples. Expression data of these proteins were derived from proteomic data.

**P* < 0.05, ***P* < 0.01, ****P* < 0.001, compared to normal adjacent tissues using Wilcoxon rank-sum test.


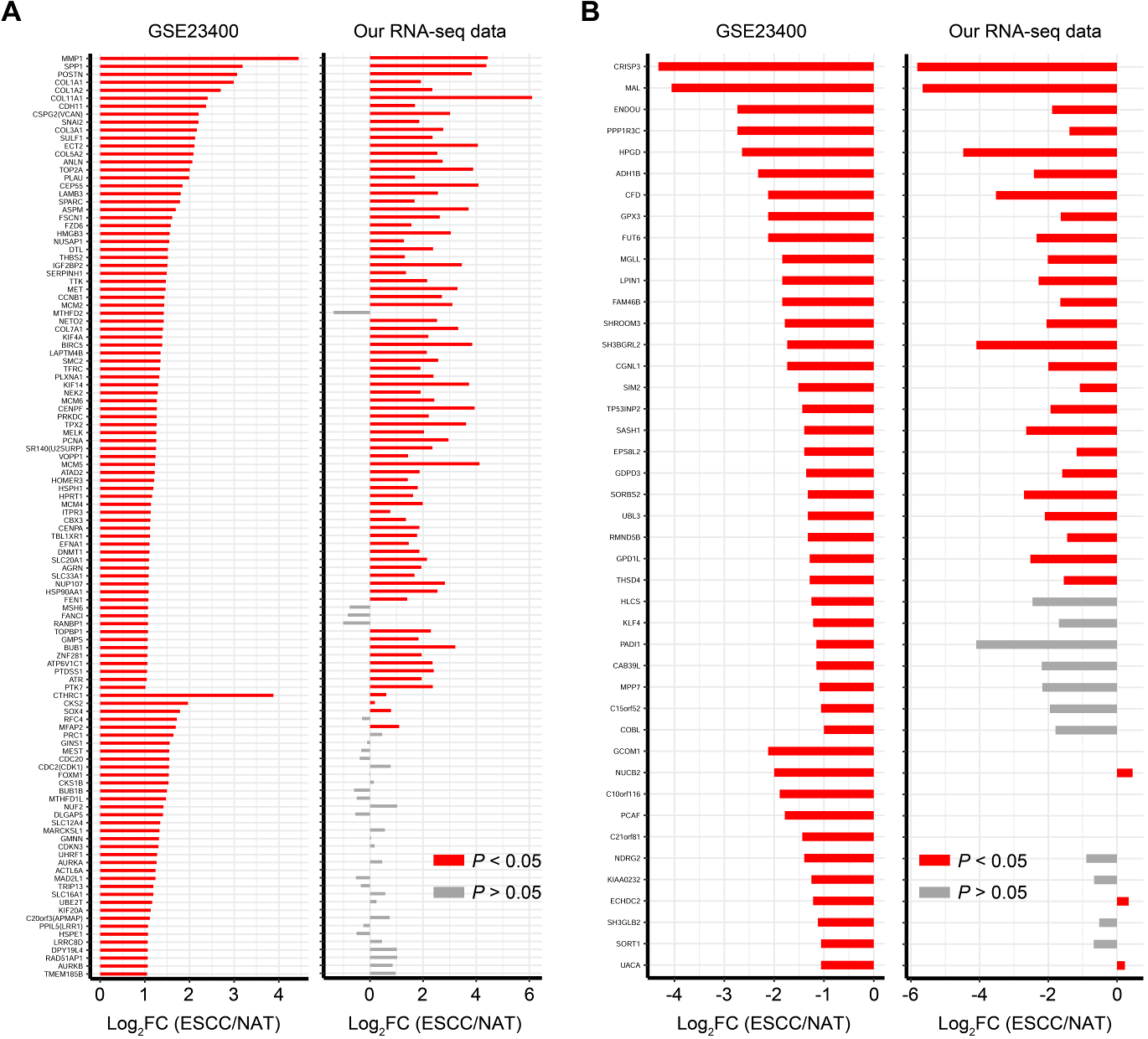


**Figure S5. Consistency analysis between the published gene expression profiling data and our RNA-seq data of ESCC patients.**

(**A**) Bar plots showing that, among those reported 116 genes with extremely upregulated expression in the public dataset GSE23400, 76 of them (65.52%) were remarkably increased in ESCC tumor tissues of our patient cohort (n = 24). (**B**) Bar plots exhibiting that, among those reported 43 genes with strikingly downregulated expression in the public dataset GSE23400, 32 of them (74.41%) were markedly repressed in ESCC tumor tissues of our patient cohort (n = 24).


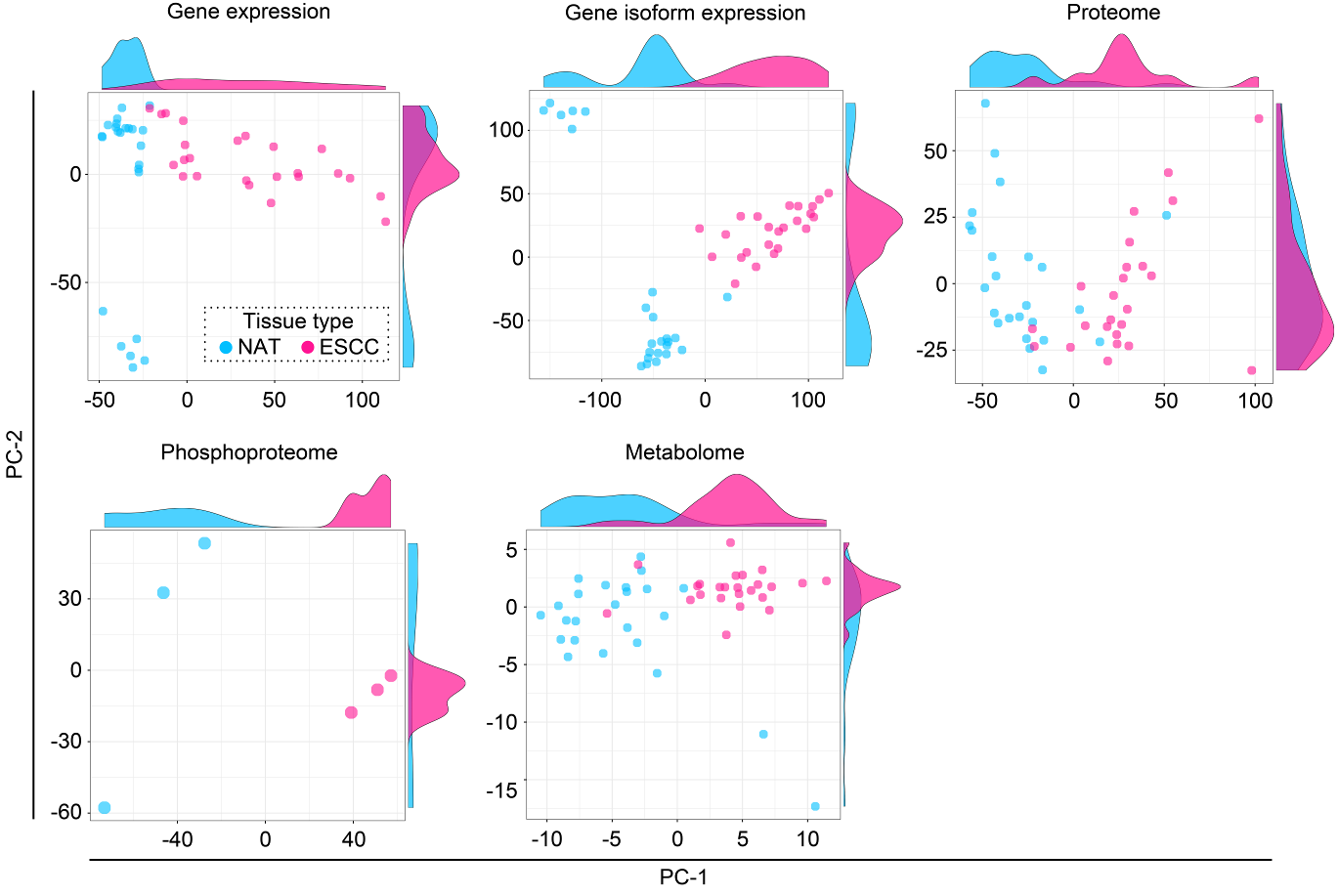


**Figure S6. Unsupervised principal component analysis of multi-omics data of ESCC patient cohort 1.** The density plots of principal component 1 (PC-1) and principal component 2 (PC-2) were displayed on the top and right-hand sides of the PCA score plots, respectively.


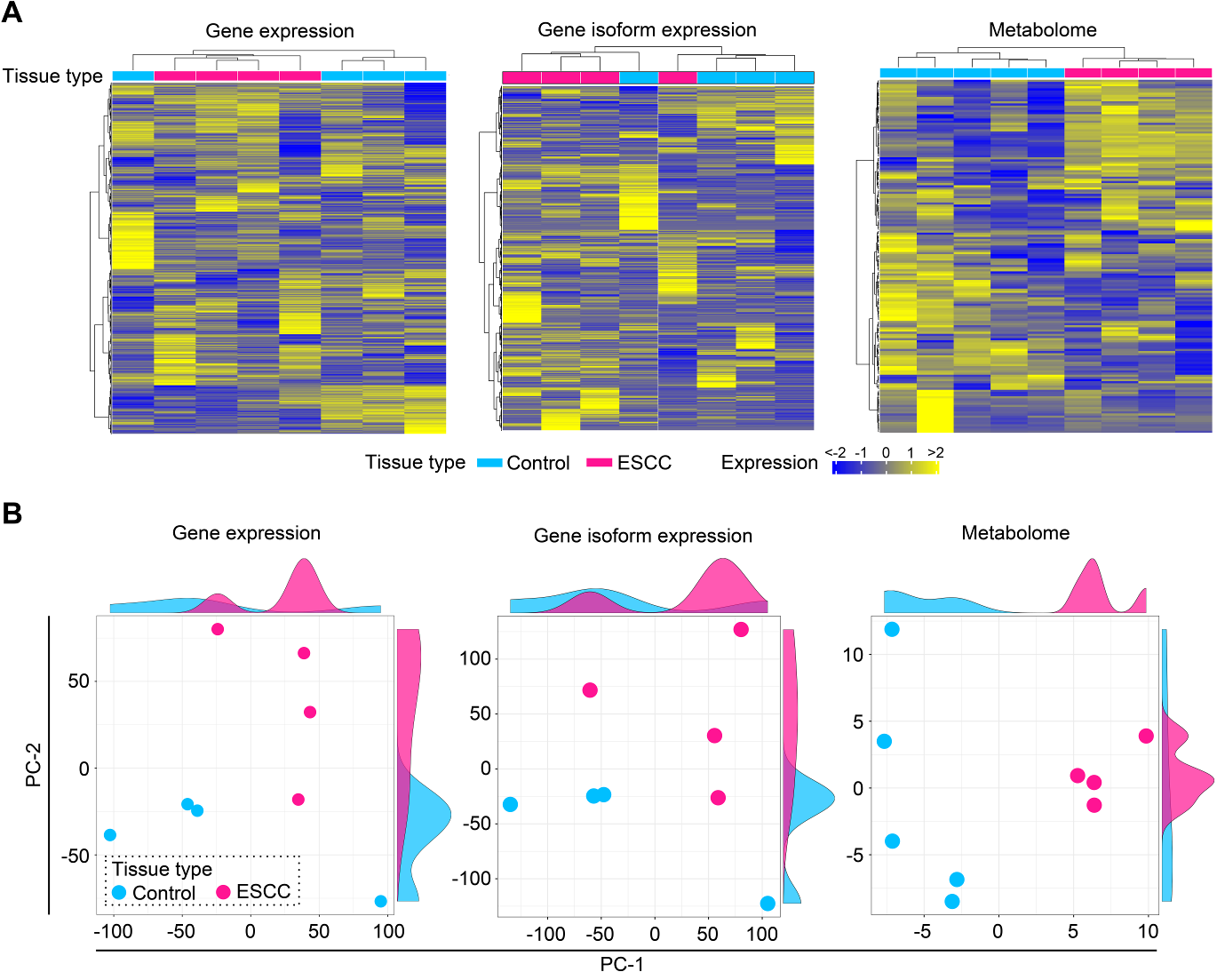


**Figure S7. RNA-seq and metabolomic analysis of esophageal tissues from ESCC and control mice.**

(**A**) Unsupervised hierarchical clustering of multi-omics data. (**B**) Unsupervised PCA analysis of multi-omics data. The density plots of PC-1 and PC-2 were displayed on the top and right-hand sides of the PCA score plots, respectively.


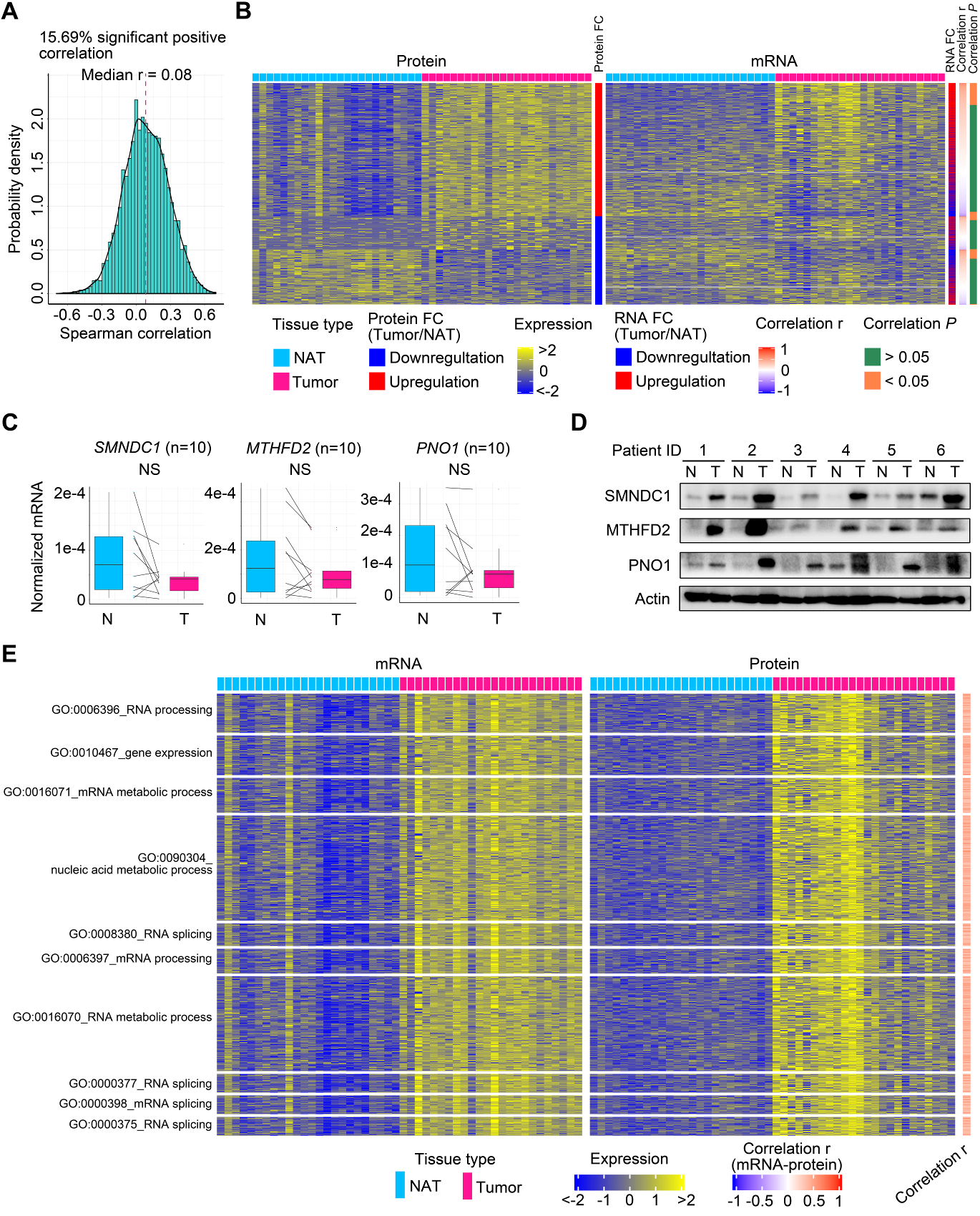


**Figure S8. The relationship between transcriptome and proteome of tissue samples of ESCC patient cohort 1.**

(**A**) Density plot of Spearman correlation coefﬁcient r values between mRNAs and corresponding proteins across 48 tissue samples. The median r was 0.08. There were 15.69% mRNAs with significant positive correlation to their corresponding proteins. (**B**) For 6,174 mRNA-protein pairs, heat maps displaying the expression of proteins as well as their corresponding mRNAs in ESCC tumors *versus* NATs. (**C**) mRNA transcription of *SMNDC1*, *MTHFD2* and *PNO1* by RT-qPCR examination. *P* values were computed using Wilcoxon rank-sum test. NS, no significance. (**D**) Protein abundance of SMNDC1, MTHFD2 and PNO1 by western blotting measurement. (**E**) Heat maps illustrating the mRNA and corresponding protein abundances of 480 genes with high transcriptional and translational activities in ESCC tumors. Of note, these mRNAs and corresponding proteins were arranged according to GO categories. T, ESCC tumor tissues; N, paired normal adjacent tissues.


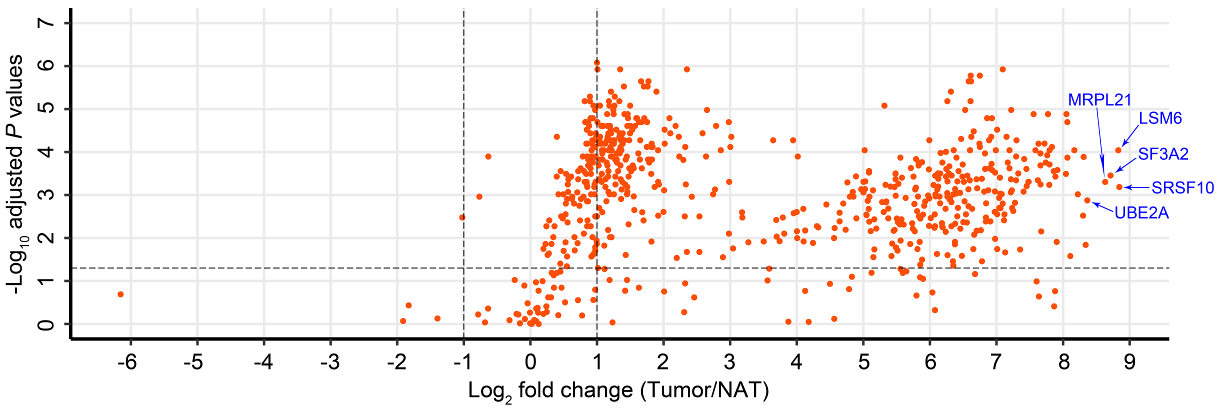


**Figure S9. Differentially expressed proteins between ESCC and NAT tissues involved in gene sets of transcriptional, post-transcriptional, translational and post-translational modulation.**

Volcano plot illustrating the fold change values (log_2_ transformed) and *P* values (-log_10_ transformed) of differentially expressed proteins between ESCC and NAT samples involved in pathways shown by Figure 3A. *P* values of proteins were obtained from the non-parametric and paired two-class Wilcoxon rank-sum test with Bonferroni correction by comparing ESCC and NAT samples.


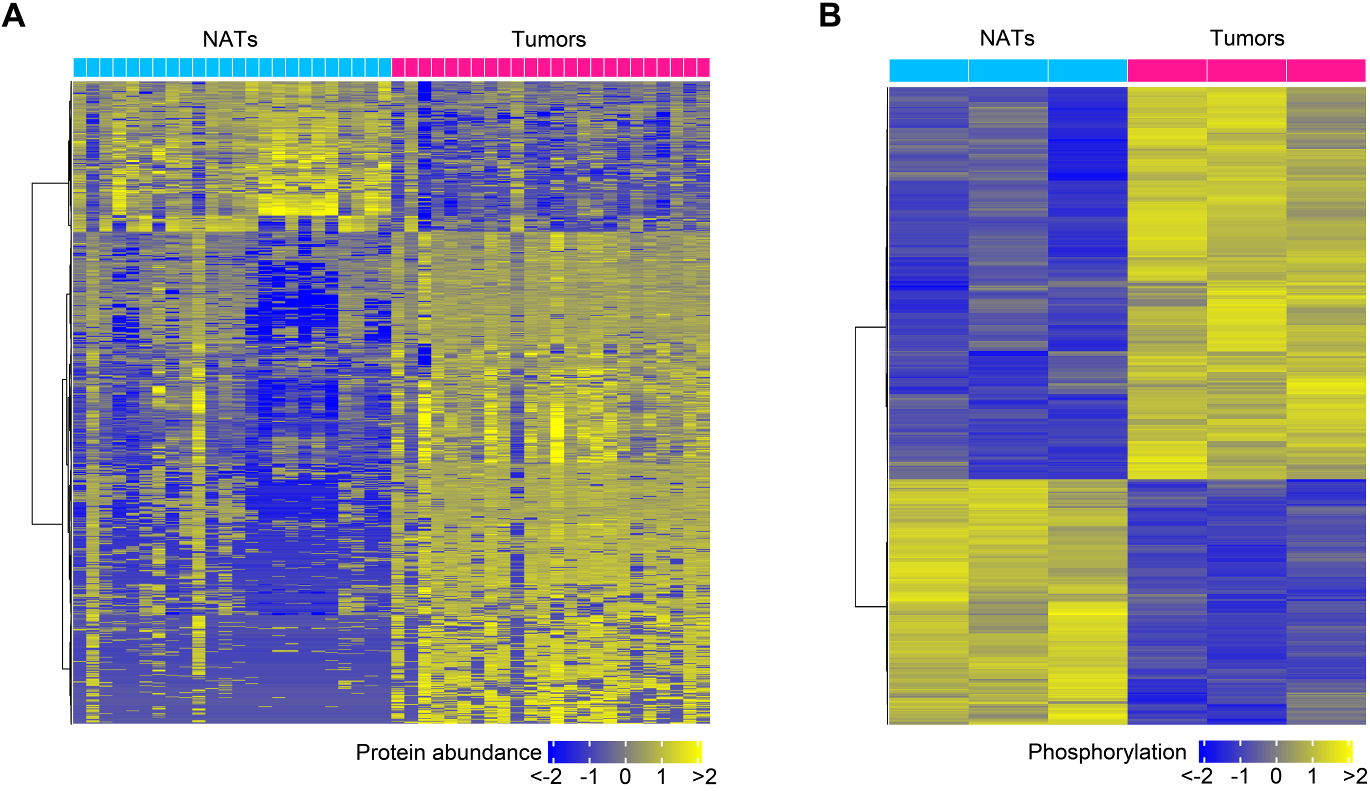


**Figure S10.** Differentially expressed proteins and phosphosites in human ESCC.

(**A**) Differentially expressed proteins (n = 2,890) between ESCC tumors and NATs (Adjusted *P* < 0.05, FDR q < 0.05, FC cutoff as 1.5) as exhibited by the heat map. (**B**) Differentially expressed phophosites (n = 517) between ESCC tumors and NATs (Adjusted *P* < 0.05, FC cutoff as 1.5) as shown by the heat map.


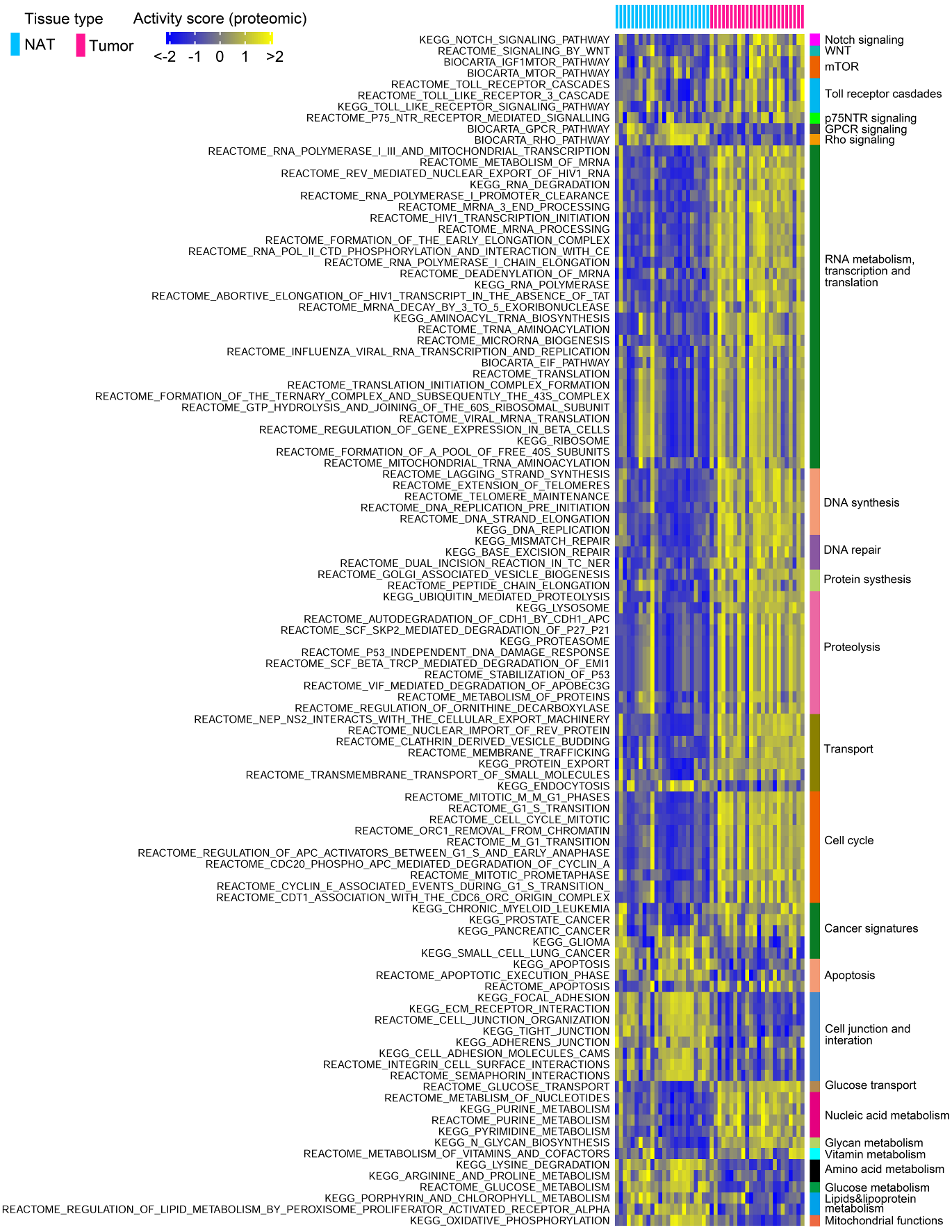


**Figure S11. Altered pathway activity of ESCC tumors inferred by proteomic data.**

Apart from the top 50 perturbed pathways showed in Figure 4B, the remaining 107 pathways with altered activity (Adjusted *P* < 0.05) between ESCC and NAT tissues were showed here.


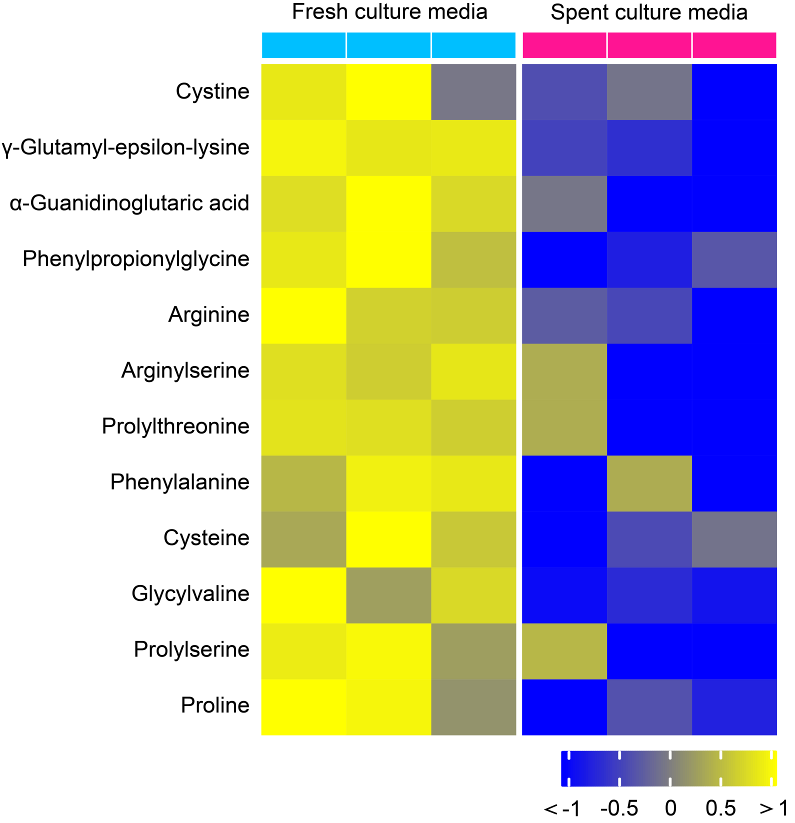


**Figure S12. Consumption of amino acids by ESCC cell line KYSE150.**

Heat map showing the changes of amino acids in spent culture media of KYSE150 cells as relative to fresh culture media. Remarkable decline of a total of 12 amino acids in the spent culture media indicated that KYSE150 cells readily imported and consumed plenty of extracellular amino acids.


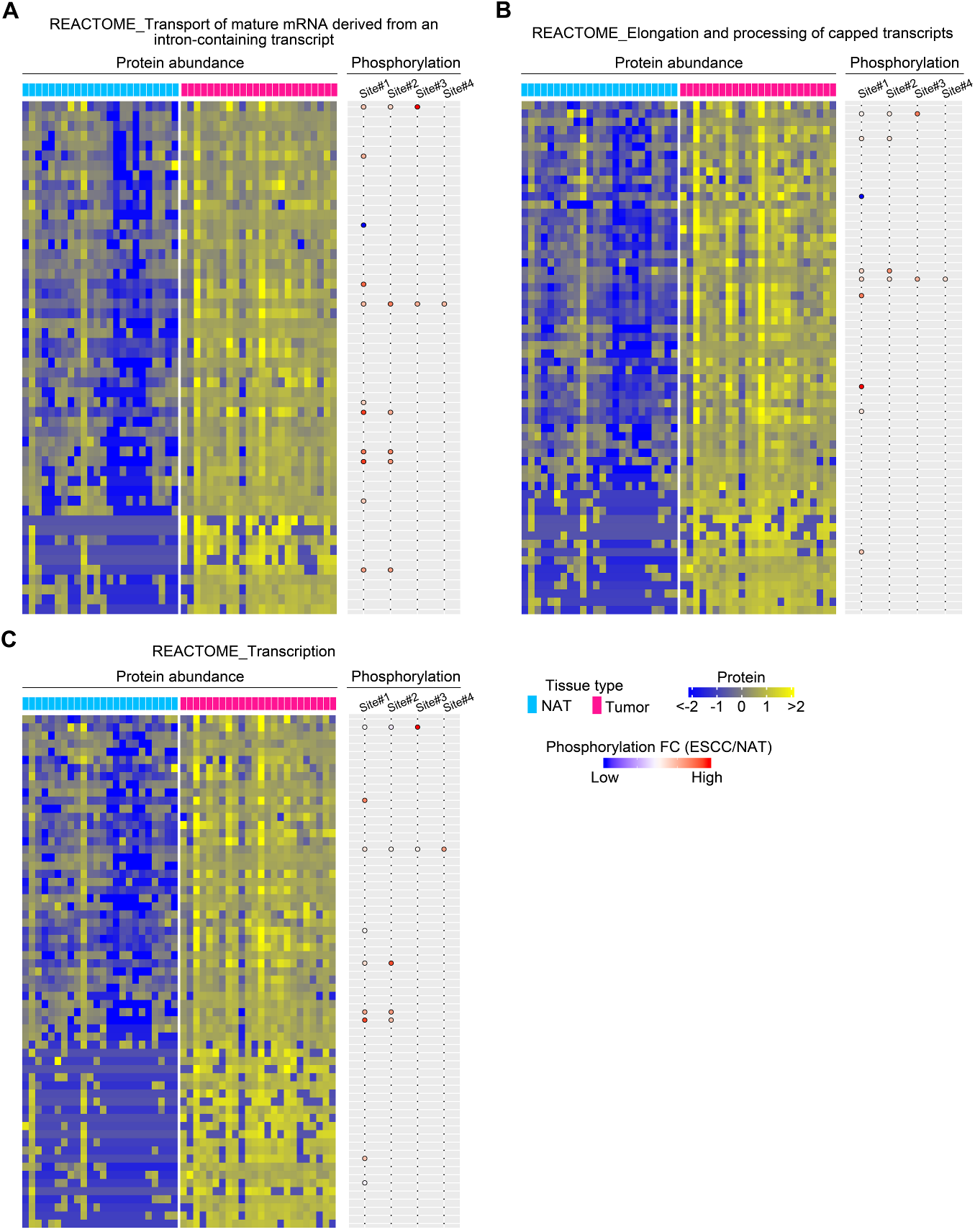


**Figure S13. Crucial pathways of ESCC inferred by the integrative analysis of proteomic and phosphoproteomic data.**

(**A-C**) Heat maps showing differential proteins between ESCC tumors and NATs in the enriched pathways, including transport of mature mRNA derived from an intron-containing transcript (REACTOME), elongation and processing of capped transcripts (REACTOME) and transcription (REACTOME). Notably, balloon plots exhibiting differential phosphosites of proteins in the matched heat maps on the left.


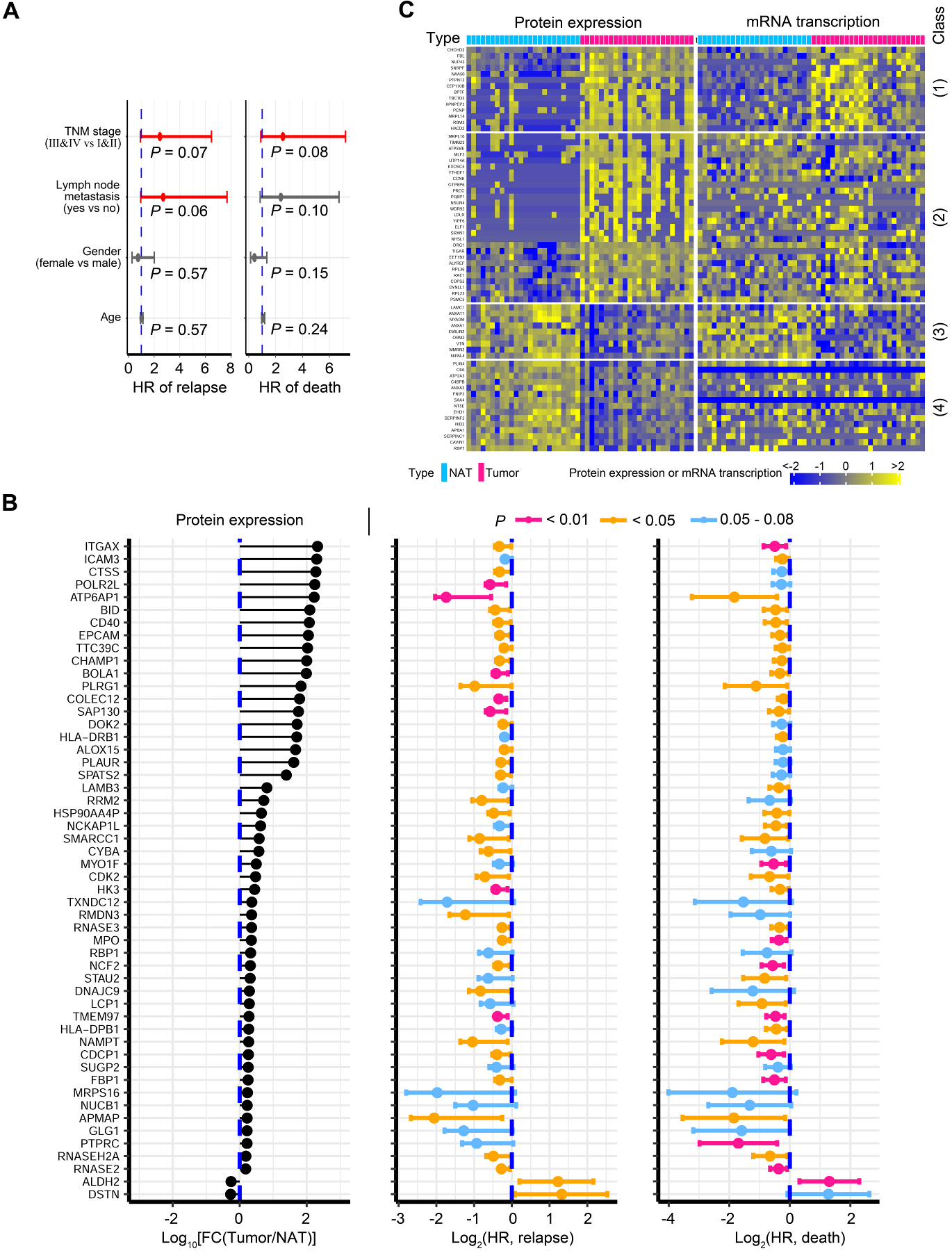
**Figure S14. Prognostic analysis of established clinical parameters and identiﬁcation of new prognostic proteins using proteomic data.**

(**A**) Univariate Cox analysis of HRs of disease relapse and death of reported clinical factors in ESCC patient cohort 1. The 95% confidential intervals of HRs were given in the figure. (**B**) A total of 52 DEPs with negative correlation to HRs of disease relapse and death were identified. For these prognostic proteins, their 95% confidential intervals of HRs of relapse or death were illustrated. HRs > 1 corresponded to an increased risk of death/relapse compared with the lower abundance of proteins, while HRs < 1 correspond to a reduced risk of death/relapse compared with the lower abundance of proteins. (**C**) Heat maps showing 66 prognostic proteins with positive correlation to HRs of disease relapse and death, as well as their corresponding mRNA between ESCC tumors and NATs. Class (1), 14 prognostic proteins exhibited consistent increased expression direction with their corresponding mRNAs in ESCC tumors relative to NATs; class (2), 28 prognostic proteins were significantly raised in ESCC tumors, whereas their corresponding mRNAs were reversely expressed or not altered in ESCC tumors; class (3), nine prognostic proteins exhibited consistent reduced expression direction with their corresponding mRNAs in ESCC tumors as relative to NATs; class (4), 15 prognostic proteins were significantly downregulated in ESCC tumors, whereas their corresponding mRNAs were not altered in ESCC tumors.


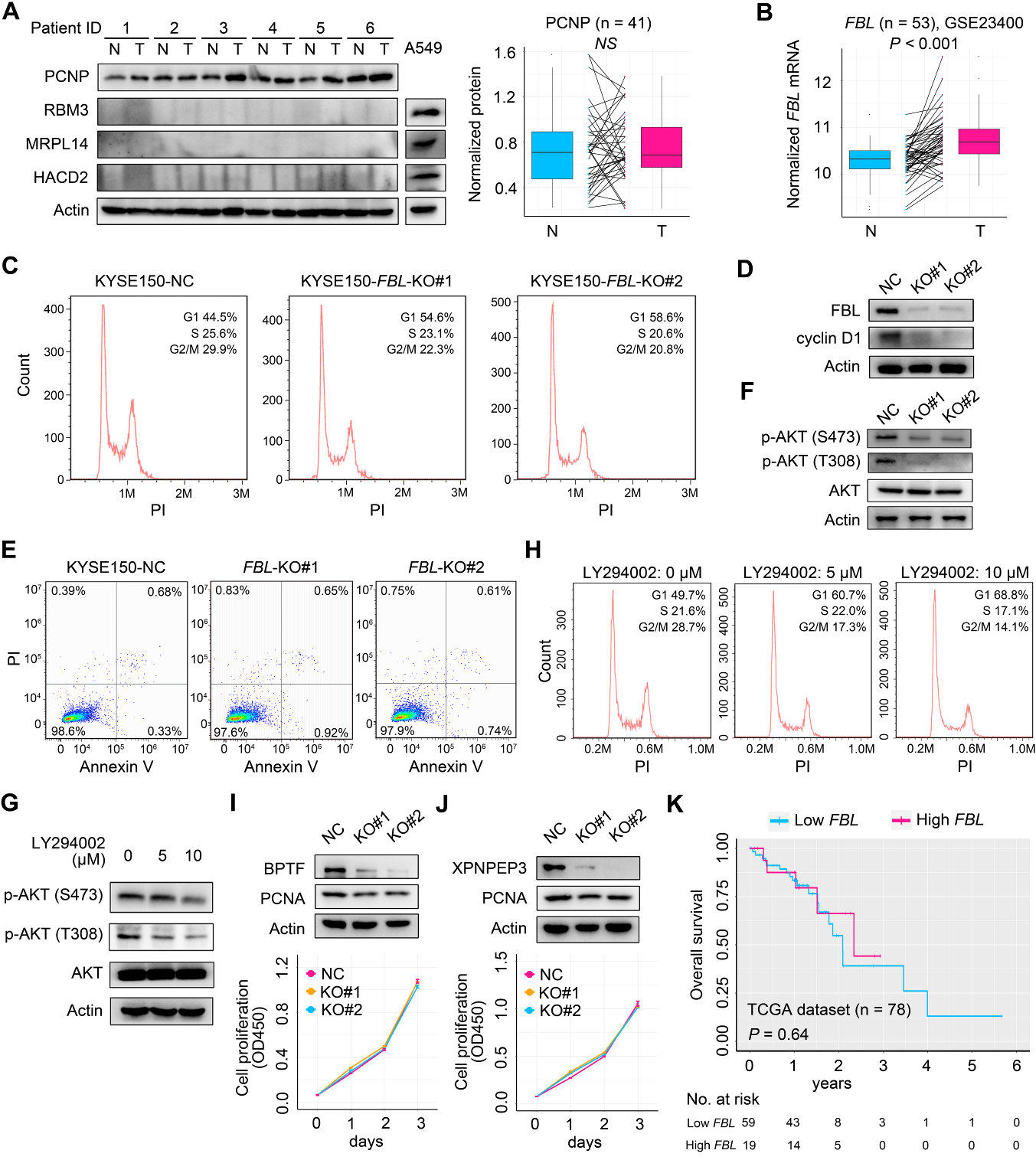


**Figure S15. Validation and functional assays of prognostic protein markers.**

(**A**) Validation for the expression of PCNP, RBM3, MRPL14 and HACD2 in ESCC tissues and NATs. The specimens were acquired from patient cohort 2. Representative western blot images were showed. The results demonstrated that RBM3, MRPL14 and HACD2 were not expressed in ESCC tissues, while PCNP was not altered. Lung cancer cell line A549 was used as a positive control for the expression of RBM3, MRPL14 and HACD2. *P* values were computed using Wilcoxon rank-sum test. NS, no significance. (**B**) The transcription of *FBL* between ESCC tissues and NATs of patients (n = 53) derived from a public data set GSE23400. *P* values were computed using Wilcoxon rank-sum test. (**C**) Cell cycle analysis of KYSE150 control cells (NC) and KYSE150 cells with *FBL* deletion (KO#1 and KO#2). (**D**) Reduced expression of cyclin D1 key for G1/S transition in KYSE150 cells with *FBL* ablation. (**E**) Cell apoptosis analysis of KYSE150 control cells and KYSE150 cells with *FBL* abrogation. (**F**) Downregulation of phosphorylated AKT at Ser473 or Thr307 caused by *FBL* deletion in KYSE150 cells. (**G**) Decline of phosphorylated AKT at Ser473 or Thr307 induced by treatment of a PI3K inhibitor LY294002 in KYSE150 cells. (**H**) Cell cycle analysis of KYSE150 cells treated with distinct concentrations of LY294002. (**I**) Deletion of *BPTF* in ESCC cell line KYSE150 did not modify PCNA expression and cell growth. (**J**) Deletion of *XPNPEP3* in ESCC cell line KYSE150 did not alter PCNA expression and cell growth. (**K**) Overall survival curves of ESCC patients with low and high *FBL* mRNA values. This dataset was derived from TCGA database.

**Table S1. RNA quantification and quality assurance by the NanoDrop ND-1000 spectrophotometer (Thermo Scientific) for samples of ESCC patient cohort 1**.

| Sample name | OD260/280 ratio^†^ | Concentration (ng/µL) |
| --- | --- | --- |
| E1N | 1.87 | 1263.6 |
| E2N | 1.86 | 1742.42 |
| E3N | 1.87 | 1764.72 |
| E4N | 1.85 | 1909.76 |
| E5N | 1.85 | 1609.73 |
| E6N | 1.82 | 1420.20 |
| E7N | 1.89 | 1786.20 |
| E8N | 1.81 | 1471.79 |
| E9N | 1.80 | 896.63 |
| E10N | 1.85 | 580.72 |
| E11N | 1.85 | 1354.60 |
| E12N | 1.89 | 1209.89 |
| E13N | 1.85 | 1999.62 |
| E14N | 1.85 | 1835.76 |
| E15N | 1.85 | 111.94 |
| E16N | 1.83 | 104.17 |
| E17N | 1.80 | 107.72 |
| E18N | 1.90 | 186.87 |
| E19N | 1.92 | 167.57 |
| E20N | 1.80 | 142.27 |
| E21N | 1.87 | 138.80 |
| E22N | 1.88 | 282.16 |
| E23N | 1.89 | 107.58 |
| E24N | 1.86 | 163.27 |
| E1T | 1.87 | 1418.85 |
| E2T | 1.87 | 2245.32 |
| E3T | 1.85 | 1233.98 |
| E4T | 1.84 | 1540.33 |
| E5T | 1.90 | 2070.68 |
| E6T | 1.81 | 1955.90 |
| E7T | 1.81 | 1395.80 |
| E8T | 1.88 | 1780.03 |
| E9T | 1.83 | 1528.60 |
| E10T | 1.86 | 1593.17 |
| E11T | 1.84 | 2581.50 |
| E12T | 1.87 | 1734.99 |
| E13T | 1.86 | 1618.18 |
| E14T | 1.87 | 1800.26 |
| E15T | 1.83 | 153.16 |
| E16T | 1.85 | 108.86 |
| E17T | 1.83 | 101.84 |
| E18T | 1.90 | 196.03 |
| E19T | 1.81 | 286.58 |
| E20T | 1.99 | 153.30 |
| E21T | 1.82 | 126.94 |
| E22T | 1.81 | 92.99 |
| E23T | 1.83 | 109.33 |
| E24T | 1.85 | 99.28 |

^†^For spectrophotometer, the OD A260/A280 ratio should be close to 2.0 for pure RNA, while ratios between 1.8 and 2.1 are acceptable.

**Table S2. Sequencing library of RNA samples of ESCC patients in cohort 1 was determined by Agilent 2100 Bioanalyzer using the Agilent DNA 1000 chip kit (Agilent, part # 5067-1504).**

| Sample name | Size (bp) | Concentration (nmol/L) | Volume (μL)^†^ | Total amount (ng) |
| --- | --- | --- | --- | --- |
| E1N | 278 | 37.1 | 10 | 68.0 |
| E2N | 275 | 41.7 | 10 | 75.6 |
| E3N | 278 | 26.0 | 10 | 47.6 |
| E4N | 273 | 26.1 | 10 | 47.1 |
| E5N | 274 | 35.0 | 10 | 63.2 |
| E6N | 277 | 39.7 | 10 | 72.6 |
| E7N | 275 | 30.6 | 10 | 55.5 |
| E8N | 274 | 42.0 | 10 | 76.0 |
| E9N | 275 | 36.0 | 10 | 65.3 |
| E10N | 275 | 43.5 | 10 | 78.8 |
| E11N | 275 | 37.8 | 10 | 68.7 |
| E12N | 271 | 34.6 | 10 | 61.9 |
| E13N | 299 | 31.8 | 10 | 62.7 |
| E14N | 301 | 28.7 | 10 | 57.1 |
| E15N | 274 | 16.6 | 10 | 30.2 |
| E16N | 294 | 18.3 | 10 | 35.6 |
| E17N | 273 | 29.1 | 10 | 52.5 |
| E18N | 290 | 21.5 | 10 | 41.4 |
| E19N | 275 | 27.6 | 10 | 50.0 |
| E20N | 274 | 18.0 | 10 | 32.5 |
| E21N | 317 | 18.1 | 10 | 37.9 |
| E22N | 307 | 20.6 | 10 | 41.6 |
| E23N | 300 | 17.6 | 10 | 34.8 |
| E24N | 309 | 14.3 | 10 | 29.1 |
| E1T | 310 | 36.8 | 10 | 75.3 |
| E2T | 306 | 28.8 | 10 | 58.1 |
| E3T | 299 | 22.2 | 10 | 43.8 |
| E4T | 278 | 23.8 | 10 | 43.7 |
| E5T | 279 | 30.2 | 10 | 55.7 |
| E6T | 277 | 17.1 | 10 | 31.2 |
| E7T | 277 | 49.0 | 10 | 89.5 |
| E8T | 307 | 38.5 | 10 | 78.2 |
| E9T | 292 | 43.1 | 10 | 83.1 |
| E10T | 284 | 24.4 | 10 | 45.8 |
| E11T | 280 | 24.9 | 10 | 46.1 |
| E12T | 275 | 34.1 | 10 | 61.8 |
| E13T | 278 | 31.6 | 10 | 58.0 |
| E14T | 277 | 36.1 | 10 | 66.1 |
| E15T | 299 | 19.6 | 10 | 38.7 |
| E16T | 302 | 21.7 | 10 | 43.2 |
| E17T | 286 | 32.5 | 10 | 61.3 |
| E18T | 277 | 14.9 | 10 | 27.2 |
| E19T | 293 | 10.5 | 10 | 20.2 |
| E20T | 304 | 13.1 | 10 | 26.2 |
| E21T | 315 | 13.3 | 10 | 27.7 |
| E22T | 300 | 8.9 | 10 | 17.7 |
| E23T | 302 | 8.8 | 10 | 17.5 |
| E24T | 303 | 11.5 | 10 | 23.0 |

^†^The libraries were adjusted to 10 nM before cluster generation.

**Table S3. Basic characteristics of ESCC patients from cohort 2.**

| Variable | ESCC (n = 41) |
| --- | --- |
| Age, years |  |
| Median | 70 |
| Range | 49-80 |
| Gender, no. (%) |  |
| Male | 25 (61) |
| Female | 16 (39) |
| WBC,10^9^/L |  |
| Medium | 5.5 |
| Range | 2.9-11.0 |
| ALT, U/L |  |
| Medium | 16.5 |
| Range | 8.0-121.0 |
| AST, U/L |  |
| Medium | 26.0 |
| Range | 17.0-136.0 |
| Creatinine, μmol/L |  |
| Medium | 67.5 |
| Range | 45.0-336.0 |
| Hepatic or renal function, no. (%) |  |
| Normal | 33 (80.5) |
| Abnormal | 3 (7.3) |
| Not available | 5 (12.2) |
| TNM stage, no. (%) |  |
| Ⅰ | 8 (19.5) |
| Ⅱ | 14 (34.1) |
| Ⅲ | 12 (29.3) |
| VI | 2 (4.9) |
| Unclassified | 5 (12.2) |
| Tumor grade, no. (%) |  |
| 1 | 11 (26.8) |
| 2 | 9 (22) |
| 3 | 10 (24.4) |
| Unclassified | 11 (26.8) |
| Lymph node metastasis, no. (%) |  |
| Positive | 18 (43.9) |
| Negative | 18 (43.9) |
| Not available | 5 (12.2) |

Hepatic abnormality as defined by ALT > 2.5 × normal value or AST > 2.5 × normal value, while renal abnormality as defined by creatinine > 2.5 × normal value.

**Table S4. Basic characteristics of ESCC patients from cohort 3.**

| Variable | ESCC (n = 100) |
| --- | --- |
| Age, years |  |
| Median | 63 |
| Range | 41-79 |
| Gender, no. (%) |  |
| Male | 80 (80.0) |
| Female | 20 (20.0) |
| TNM stage, no. (%) |  |
| Ⅰ | 32 (32.0) |
| Ⅱ | 43 (43.0) |
| Ⅲ | 16 (16.0) |
| VI | 7 (7.0) |
| Unclassified | 2 (2.0) |
| Lymph node metastasis, no. (%) |  |
| Positive | 24 (24.0) |
| Negative | 76 (76.0) |
| Death, yes or no. (%) |  |
| Yes | 85 (85.0) |
| No | 15 (15.0) |
| Relapse, yes or no. (%) |  |
| Yes | 11 (11.0) |
| No | 89 (89.0) |
